# Harnessing Natural Diversity to Probe Metabolic Pathways

**DOI:** 10.1371/journal.pgen.0010080

**Published:** 2005-12-30

**Authors:** Oliver R Homann, Houjian Cai, Jeffrey M Becker, Susan L Lindquist

**Affiliations:** 1 Committee on Genetics, University of Chicago, Chicago, Illinois, United States of America; 2 Whitehead Institute for Biomedical Research, Cambridge, Massachusetts, United States of America; 3 Department of Microbiology, University of Tennessee, Knoxville, Tennessee, United States of America; Washington University in St. Louis, United States of America

## Abstract

Analyses of cellular processes in the yeast *Saccharomyces cerevisiae* rely primarily upon a small number of highly domesticated laboratory strains, leaving the extensive natural genetic diversity of the model organism largely unexplored and unexploited. We asked if this diversity could be used to enrich our understanding of basic biological processes. As a test case, we examined a simple trait: the utilization of di/tripeptides as nitrogen sources. The capacity to import small peptides is likely to be under opposing selective pressures (nutrient utilization versus toxin vulnerability) and may therefore be sculpted by diverse pathways and strategies. Hitherto, dipeptide utilization in *S. cerevisiae* was solely ascribed to the activity of a single protein, the Ptr2p transporter. Using high-throughput phenotyping and several genetically diverse strains, we identified previously unknown cellular activities that contribute to this trait. We find that the Dal5p allantoate/ureidosuccinate permease is also capable of facilitating di/tripeptide transport. Moreover, even in the absence of Dal5p and Ptr2p, an additional activity—almost certainly the periplasmic asparaginase II Asp3p—facilitates the utilization of dipeptides with C-terminal asparagine residues by a different strategy. Another, as-yet-unidentified activity enables the utilization of dipeptides with C-terminal arginine residues. The relative contributions of these activities to the utilization of di/tripeptides vary among the strains analyzed, as does the vulnerability of these strains to a toxic dipeptide. Only by sampling the genetic diversity of multiple strains were we able to uncover several previously unrecognized layers of complexity in this metabolic pathway. High-throughput phenotyping facilitates the rapid exploration of the molecular basis of biological complexity, allowing for future detailed investigation of the selective pressures that drive microbial evolution.

## Synopsis

Model organisms have allowed researchers to characterize basic biological processes in exquisite detail. Homann et al. suggest that this knowledge base presents a unique opportunity to exploit the natural genetic variation found in diverse isolates of the same species to gain new insights. Using high-throughput technology to assay growth, Homann et al. found that laboratory strains, vineyard isolates, and clinical isolates of the yeast *Saccharomyces cerevisiae* exhibit very different capacities to utilize di/tripeptides as nutrient sources. This led to the discovery of new di/tripeptide utilization activities and an elaboration of their specificities. Variations in the strength of these activities determine the spectrum of di/tripeptides that are preferentially utilized in a given strain. In turn, this influenced the vulnerability of these strains to a toxic peptide, which exploits the transport machinery to gain entry into the cell. This raises the intriguing possibility that opposing selective pressures, in which the benefit of utilizing di/tripeptides as a nutrient source is offset by the risk of importing toxic peptides, may have shaped the observed natural diversity. The natural genetic diversity present among isolates of model organisms augurs a rich resource for revealing complexities in molecular pathways and exploring the selective pressures that shaped them.

## Introduction

Our understanding of the inner workings of eukaryotic cells owes much to the yeast *Saccharomyces cerevisiae*. The application of powerful genetic and molecular tools to this model organism has yielded an extensively annotated proteome. These analyses have benefited greatly from the engineering of experimentally tractable strains of *S. cerevisiae,* but an unintended consequence of this focus has been a tendency to ignore the vast wealth of natural genetic variation found in diverse strains of this organism. Following the “gold rush” ushered in by the sequencing of the *S. cerevisiae* genome, efforts are being made to revisit this natural diversity. Phenotypic analyses of diverse yeast strains [1,2] and the application of microarray technology to the analysis of allelic variation [3–8] and population genetic variation in gene expression [9–11] are providing new insights into the ecology and diversity of the species. Such analyses should also be applicable to the elucidation of pathways that face strong diversifying selection, such as those governing uptake and metabolism of nutrients. As a test case, we employed high-throughput phenotyping of diverse *S. cerevisiae* strains to dissect the multiple activities contributing to the utilization of di/tripeptides as a nitrogen source.

The capacity to import small peptides is a ubiquitous cellular function, found in bacteria, fungi, plants, and animals [12–16]. Although small peptides have clear nutritional value as a source of amino acids, carbon, and nitrogen, they can have additional beneficial functions. For example, studies in bacteria have demonstrated a link between peptide transport and chemotaxis [17], sporulation [18,19], and the recycling of cell wall peptides [20].

Peptide transport systems, however, can also be a source of biological vulnerability. A variety of antimicrobial and antifungal agents utilize the di/tripeptide transport machinery to gain entry into the cell [21,22]. Thus, the benefits and risks associated with peptide import represent conflicting evolutionary pressures that may help shape the regulation and substrate specificity of peptide transporters. In a similar vein, transporters of small peptides are also of great interest for their medical applications, as routes for delivery of peptidomimetic drugs [23,24].

Members of the PTR family of peptide transporters transport a variety of substrates, including nitrates, amino acids, and di/tripeptides [12,25]. All PTR members contain 12 predicted transmembrane helices, and transport a variety of substrates by means of proton-motive force [26,27]. Although many organisms, such as humans and plants, contain multiple PTR2 family members, the yeast *S. cerevisiae* contains only one, the di/tripeptide transporter Ptr2p.

**Figure 1:**
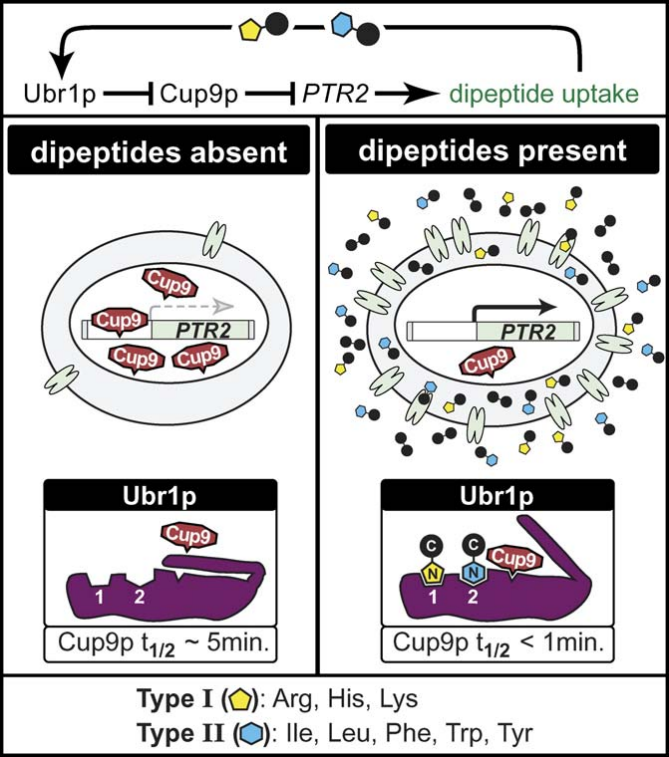
Di/Tripeptide Uptake Is Enhanced by a Positive Feedback Loop Governed by the N-End Rule Pathway The E3 ubiquitin ligase Ubr1p contains binding sites for proteins containing Type 1 (Arg, His, and Lys) and Type 2 (Ile, Leu, Phe, Trp, and Tyr) N-terminal amino acids. In the absence of di/tripeptides, the *PTR2* repressor Cup9p is abundant and *PTR2* expression is minimal. Upon binding of Type 1 and/or Type 2 di/tripeptides to Ubr1p, an autoinhibitory domain blocking the recognition of an internal Cup9p degron dissociates from the Cup9p binding site, decreasing the half-life of Cup9p (t _½_) from approximately 5 min to less than 1 min. Consequently, *PTR2* repression is relieved and elevated levels of Ptr2p enhance di/tripeptide uptake, initiating a positive feedback loop. This figure was adapted from the diagram of Turner et al. [33], with the authors' permission.

The regulation of *PTR2* expression in *S. cerevisiae* is strongly influenced by the composition of the extracellular environment. *PTR2* expression is induced in the absence of preferred nitrogen sources [28]—even more so when the medium contains particular amino acids [29]. Import of di/tripeptides containing basic or bulky hydrophobic N-terminal residues also induces *PTR2* by reducing cellular levels of the *PTR2* repressor Cup9p. Specifically, these di/tripeptides serve both as ligands and as regulators of the E3 ubiquitin ligase Ubr1p, which mediates a protein degradation system that is governed by the identity of N-terminal amino acids [30,31] (Figure 1). These peptides are too small to serve as targets for degradation by the proteasome, and they are assimilated as nutrients via other peptidases in the cells. However, di/tripeptides with basic (Type 1: Arg, His, or Lys) and bulky (Type 2: Ile, Leu, Phe, Trp, or Tyr) N-terminal residues can compete with larger protein substrates for binding at the Type 1 and Type 2 Ubr1p substrate-binding sites. Upon binding, these peptides allosterically activate Ubr1p-mediated degradation of Cup9p by release of the Ubr1p autoinhibitory domain, thus exposing a substrate-binding domain that binds an internal degron in Cup9p [32]. Relief of Cup9p repression of *PTR2* results in enhanced *PTR2* expression. This initiates a positive regulatory feedback loop in which di/tripeptide uptake perpetuates Ubr1p-mediated degradation of Cup9p, up-regulation of *PTR2,* and additional di/tripeptide uptake [32–34].

Investigations of di/tripeptide import in *S. cerevisiae* have typically been limited to the analysis of a small number of substrates through complementation of auxotrophies or the use of radiolabeled dipeptides. The development of the Biolog (Hayward, California, United States) system of Phenotype MicroArrays (PM) [35–37] provides the opportunity to dramatically expand the scope of these analyses. The PM system facilitates the classification of bacterial and fungal species by generating a characteristic phenotypic profile, which reflects the metabolic capabilities and chemical sensitivities of different species. The strong representation of peptides in the PM assay plates—284 of the 400 possible dipeptide permutations and 11 tripeptides—provides a unique opportunity to assay the capacity of different *S. cerevisiae* strains to import and utilize dipeptides as nitrogen sources.

Using high-throughput data from PM assays, we uncovered extensive variation in dipeptide utilization in different strains of *S. cerevisiae*. We exploited this variation to uncover a diversity of strategies that may serve to balance the opposing selection pressures of nutrient utilization and toxin vulnerability. We find that Ptr2p provides only one of at least four distinct molecular activities that facilitate the utilization of dipeptides in *S. cerevisiae*. The varying strengths of these activities in different strains were key to their identification. No single strain would have revealed the full complexity of dipeptide utilization, underscoring the importance of natural genetic variation for developing a comprehensive understanding of cellular processes. We suggest that the comparison of genetically diverse strains will provide many new avenues for exploring the malleability of basic cellular functions and the evolutionary mechanisms and selective pressures that shape complex traits.

## Results

To advance our understanding of the processes contributing to nitrogen utilization in *S. cerevisiae,* we studied a panel of strains isolated from several widely differing growth environments (Table 1). We included two related laboratory strains, S288c and W303, two California vineyard isolates, RM3 and RM8, a vineyard isolate thought to be of French origin, Y55 [38], and a clinical isolate, YAT7. Because expression of the dipeptide transporter *PTR2* is strongly induced by even micromolar amounts of certain amino acids supplemented in the media [29], we first restored all of these strains to full amino acid prototrophy (see Materials and Methods).

**Table 1:**
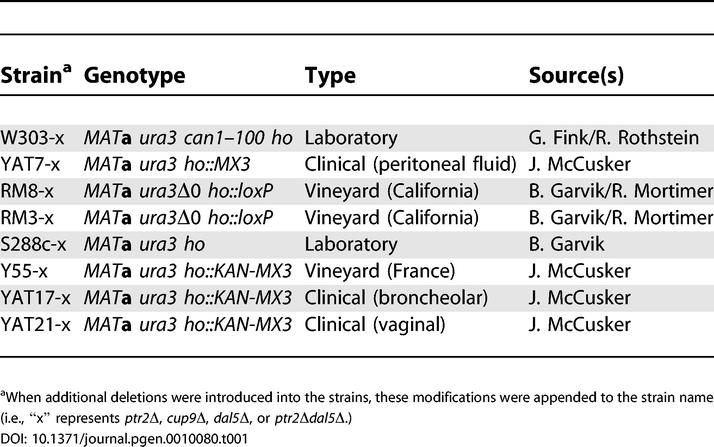
Strains Used in This Study

### Assaying Nitrogen Source Utilization in Diverse Strains Using PM Assays

We utilized the PM system to assay the ability of these strains to utilize an extensive selection of dipeptides and other compounds as nitrogen sources. Cells were grown in 96-well plates in which each well contained a different nitrogen source. A proprietary tetrazolium dye solution, which is reduced to the purple compound formazan, was included in each well. Plates were photographed every 15 min, generating a growth curve for each well that primarily reflected dye reduction [39]. The remaining growth signal was attributable to turbidity resulting from cell growth.

Because the technology was developed for bacterial cells, we first asked how well the PM signal curves reflect yeast cell growth and respiration, and how reproducible these phenotypes were in standard yeast laboratory media. Reduction of the tetrazolium dye ostensibly results from cellular respiration [39], but when we tested petite strains of *S. cerevisiae* in the PM assay, they remained capable of robust dye conversion (data not shown). Thus, mitochondrial respiration is not required to produce reductants for this dye. Next, we employed dye-independent measures of growth, such as measuring optical density of PM plates lacking the tetrazolium dye and plating serial dilutions of cells from PM plates to solid media to measure the number of colony-forming units in each PM well. Dye conversion correlated well with growth (see Materials and Methods), except that dipeptides containing the amino acids histidine, lysine, and/or cysteine sometimes caused dye reduction in the absence of growth (data not shown). Interestingly, this growth-independent dye reduction occurred only in the presence of cells. Since it reflects an unknown metabolic process, these dipeptides were omitted from our analyses. It should be noted, however, that this growth-independent dye reduction exhibited strong inter-strain variation, suggesting yet another source of metabolic variation for further study.

The PM assay utilizes a viscous medium that supports less-vigorous growth than typical laboratory media (data not shown). To determine if growth on diverse substrates in PM plates correlates well with standard laboratory media, we assayed growth on dipeptides as the sole nitrogen source in both solid and liquid standard media (see below), using optical density at 600 nm to measure growth in the liquid assays. Given the sensitivity of the dipeptide transport machinery to environmental cues, it is unsurprising that some differences were observed between these platforms. In large part, however, these differing growth conditions provided similar results, and the reproducibility on all tested media was robust.

### Shared Properties and Inter-Strain Variation in Nitrogen Source Utilization

**Figure 2:**
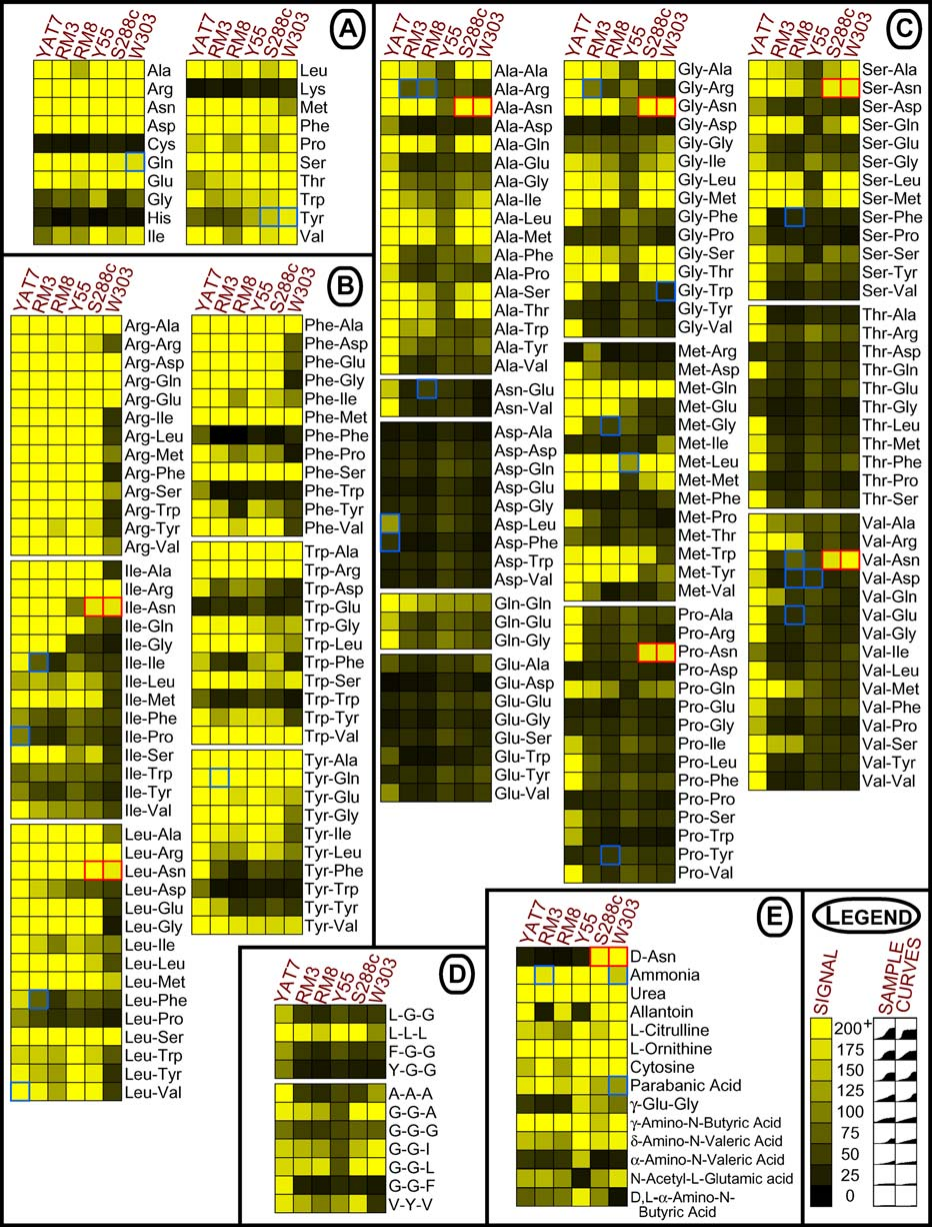
Variation in Nitrogen Source Utilization among Six Strains of *S. cerevisiae* Each square represents the growth of one strain in the PM assay supplied with the indicated nitrogen source. The value reflecting the extent of growth was generated from the 48-h signal curve of the PM assay and represented by the intensity of coloration, as shown in the legend. All signal values exceeding 200 reflected very robust growth, and are therefore represented with the same color. Blue-bordered squares denote nitrogen sources that produced varying results in that strain, exhibiting substantial variation in signal among replicates (σ^2^/mean >10 and at least one replicate with signal >50 or <200). Red-bordered squares denote putative substrates of the Asp3p asparaginase. (A) Amino acids, (B) N-end rule dipeptides, (C) non–N-end rule dipeptides, (D) tripeptides; amino acids are represented as single letters, and (E) miscellaneous nitrogen sources.

Next, we asked if the natural diversity in our yeast strains produced phenotypic diversity in nitrogen source utilization. The 48-h PM growth curves for each strain on each medium are provided in the supplemental materials (Dataset S1–S3). To simplify comparisons, each growth curve was assigned a score reflecting the extent of growth, as shown for representative curves in the legend of Figure 2 (see also Materials and Methods). In agreement with an earlier study of the ability of individual amino acids to serve as nitrogen sources in *S. cerevisiae* [40], we found that cysteine, histidine, and lysine did not support growth in any of our strains (Figure 2A). These are the same amino acids that, when present in dipeptides, caused some strains to reduce the tetrazolium dye in the absence of growth. Apart from some inter-strain variation in growth on glycine and tyrosine, the remaining amino acids supported robust growth in all strains tested.

In contrast to the general uniformity in the utilization of individual amino acids, the six strains we analyzed had very different patterns of dipeptide utilization. To assist visualization of the data, dipeptides were separated into two classes: N-end rule dipeptides, which up-regulate *PTR2* by enhancing Ubr1p-mediated degradation of the Cup9p repressor (Figure 2B), and non–N-end rule dipeptides (Figure 2C). The clinical isolate YAT7 utilized the broadest spectrum of dipeptides (see Figure 2C in particular) and tripeptides (Figure 2D). Indeed, it grew robustly on many peptides that none of the other strains could utilize well, if at all. (Preliminary analyses of two additional clinical isolates, YAT17 and YAT21, suggest that robust di/tripeptide transport may be a common feature of strains adapted to the atypical mammalian host environment; data not shown.) The laboratory strain W303 utilized the fewest dipeptides. It lacked the broad capacity for N-end rule dipeptide utilization characteristic of the other strains (Figure 2B), but it did grow robustly on some of these dipeptides. In contrast, growth of the French vineyard isolate Y55 was limited on the non–N-end rule dipeptides in comparison to the other strains tested (Figure 2C). Each strain also exhibited a unique pattern in the utilization of other nitrogen sources (Figure 2D and 2E).

Given the close relationship between W303 and S288c (W303 was derived in part from crosses with S288c; R. Rothstein, personal communication), it was surprising that the PM profiles of S288c more closely resembled those of the California vineyard isolates RM3 and RM8. However, S288c and W303 did share two characteristics: Both grew robustly on all of the dipeptides in the array that contained C-terminal asparagine residues (Figure 2B and 2C, boxes with red borders), and both grew well on D-asparagine (Figure 2E).

### Overexpression of *PTR2* Masks Inter-Strain Variation and Enhances Di/Tripeptide Utilization

The diversity of dipeptide-utilization traits suggests profound differences in the underlying mechanism of di/tripeptide import among our conspecific strains. One likely source of inter-strain variation in dipeptide utilization is differences in *PTR2* regulation. If dipeptide utilization is limited by low levels of Ptr2p, up-regulation of *PTR2* should enhance growth. To determine how broadly a lack of *PTR2* expression limits dipeptide utilization, we eliminated the repressor of *PTR2,* Cup9p, in three strains whose wild-type dipeptide-utilization profiles were robust, intermediate, and weak: YAT7, RM8, and W303, respectively.

**Figure 3:**
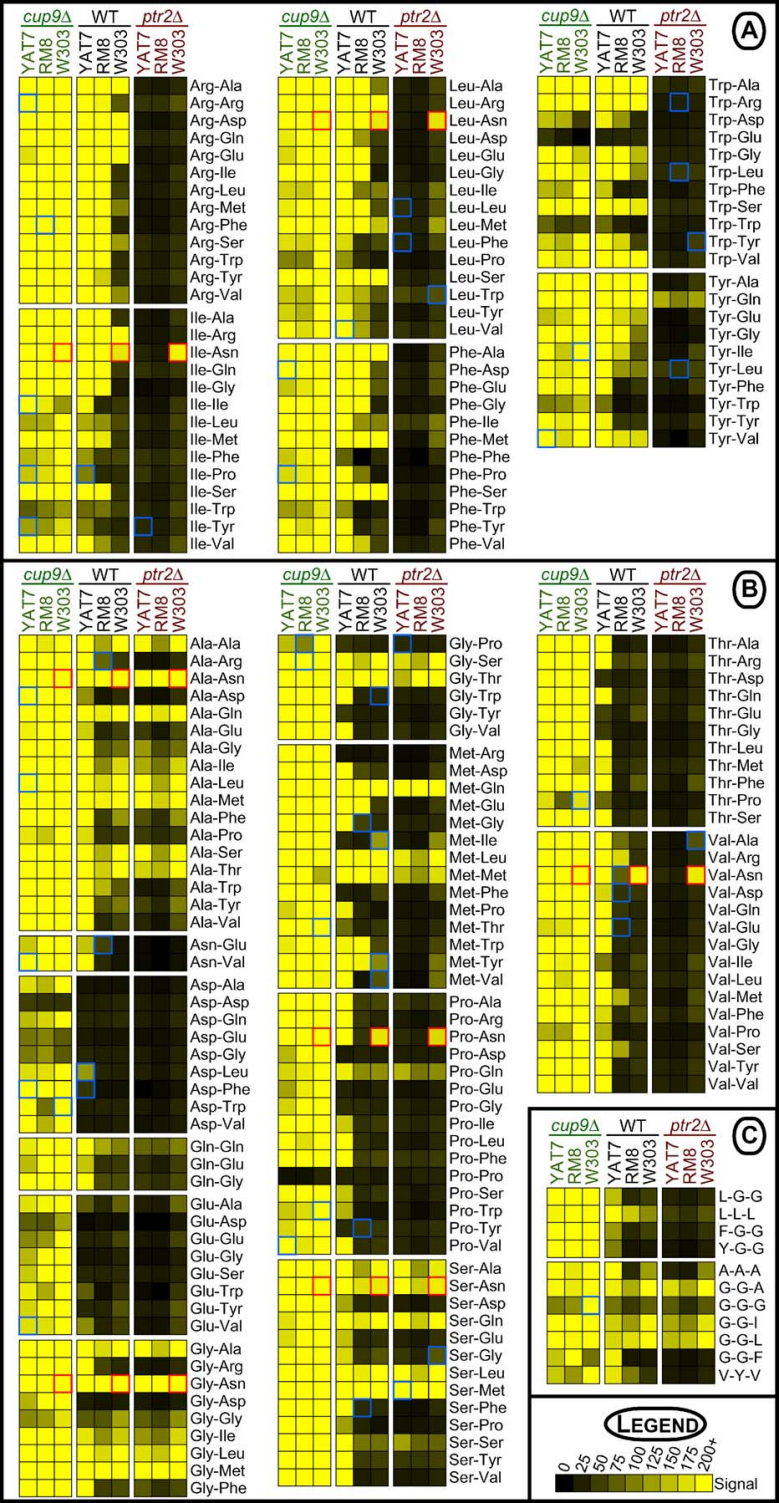
Deletion of *CUP9* and *PTR2* Strongly Impacts Di/Tripeptide Utilization in *S. cerevisiae* Strains Elimination of the *PTR2* repressor Cup9p revealed that Ptr2p can facilitate the utilization of nearly all di/tripeptides tested in the PM assay, yet Ptr2p was not required for the utilization of a subset of di/tripeptides as a nitrogen source. See Figure 2 for a detailed explanation of the yellow boxes and colored borders. (A) N-end rule dipeptides, (B) non–N-end rule dipeptides, and (C) tripeptides; amino acids are represented as single letters.

In all three strain backgrounds, the *CUP9* deletions caused robust growth on most of the di/tripeptides present in the arrays (Figure 3). Even dipeptides that had not supported growth in any of the wild-type strains, such as dipeptides bearing the N-terminal acidic residues aspartate or glutamate, supported growth upon deletion of *CUP9* (Figure 3B). Of the 197 dipeptides amenable to PM analysis, only a small fraction—most notably Trp-Trp, Trp-Glu, Asp-Asp, and Pro-Pro—supported little growth in the *cup9*Δ strains. These dipeptides may either be refractory to transport or less amenable to use as a nitrogen source following transport. The robust dipeptide utilization phenotype of the *cup9*Δ mutants indicates that Ptr2p is capable of transporting a very broad array of di/tripeptides, a property that has long been assumed but never addressed with such a large spectrum of dipeptides.

Further, the robust dipeptide-utilization phenotypes of the *cup9*Δ mutant strains indicate that differences in regulatory control of *PTR2* are a key feature of the natural diversity observed among the strain backgrounds. Whereas the wild-type strains exhibited different dipeptide-utilization phenotypes (see Figure 2B and 2C), relief of *PTR2* repression by deletion of *CUP9* eliminated almost all inter-strain variation (Figure 3). Thus, the limited capacity for dipeptide utilization in wild-type W303 relative to RM8 and YAT7 is likely to reflect a difference in *PTR2* regulation rather than a difference in Ptr2p function.

### Ptr2p Is Not Required for the Utilization of Some Dipeptides

The only known transporter of di/tripeptides in *S. cerevisiae* is Ptr2p. Therefore, we expected that *ptr2*Δ strains would be unable to utilize any di/tripeptides in the PM assay. Surprisingly, this was not the case, particularly for non–N-end rule di/tripeptides (Figure 3B and 3C). All three strains tested did not require *PTR2* to utilize a subset of these non–N-end rule dipeptides. Indeed, the W303-*ptr2*Δ mutant grew just as well as the wild-type strain on the non–N-end rule dipeptides.

When examined as a whole, these observations suggested that at least two activities contribute to dipeptide utilization: Ptr2p-mediated transport and an as-yet-unidentified activity. For N-end rule dipeptides, Ptr2p was the main activity contributing to their utilization, as evidenced by the almost complete abrogation of growth on these nitrogen sources by the *ptr2*Δ deletions (Figure 3A). For non–N-end rule di/tripeptides, both Ptr2p and the Ptr2p-independent activity played roles in their utilization (Figure 3B). The contribution of Ptr2p to general dipeptide utilization varied from minimal, as observed with W303, to substantial, as observed with the clinical isolate YAT7 (Figure 3, compare wild-type and *ptr2*Δ strains). In contrast, the similar growth properties of the *ptr2*Δ strains suggested that the contribution of the Ptr2p-independent activity was similar in all three strain backgrounds.

### Dal5p Confers a Ptr2p-Independent Dipeptide-Utilization Activity

To identify the source of dipeptide utilization in the *ptr2*Δ strains, we took advantage of the robust growth of the W303-*ptr2*Δ strain when Ala-Leu was the sole source of nitrogen in the PM assay. We speculated that the concentration of Ala-Leu needed to support growth would be reduced if the gene allowing its utilization was overexpressed. Accordingly, we screened a high-copy (2 μm) genomic library for plasmids that restored growth of W303-*ptr2*Δ on limiting quantities of Ala-Leu (0.75 mM). After eliminating candidates that contained the *PTR2* open reading frame, the three remaining plasmids were sequenced. Each plasmid carried the allantoate/ureidosuccinate permease gene *DAL5*.

**Figure 4:**
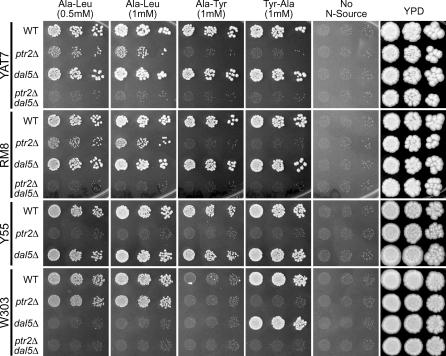
The Relative Contributions of Dal5p and Ptr2p to Dipeptide Utilization Vary among Strains of *S. cerevisiae* Strains were cultured on YPD plates, as with the PM assays, and then suspended in water at a concentration of approximately 3.5 × 10^6^ cells/ml and plated as 1×, 5×, and 25× dilutions (from left to right). Each plate contained MM plus the nitrogen source indicated at the top of the column (with the exception of the far right column, in which YPD plates were used). Plates were incubated at 30 °C for 4 d and then photographed. A Y55-*ptr2*Δ*dal5*Δ double mutant was not tested, since deletion of *PTR2* alone was sufficient to eliminate dipeptide utilization.

To analyze the contribution of Dal5p to basal dipeptide utilization, we constructed *ptr2*Δ, *dal5*Δ, and *ptr2*Δ*dal5*Δ mutants in a variety of strain backgrounds. We began by testing the growth of serially diluted cells on conventional solid media containing different nitrogen sources (Figure 4). These included the putative Dal5p substrate Ala-Leu, as well as Tyr-Ala and Ala-Tyr, which had not supported growth of the *ptr2*Δ mutant in any of the strains tested in the PM assay (see Figure 3A and 3B). In all strains tested, the *ptr2*Δ*dal5*Δ double mutant abolished growth on all three assayed dipeptide sources. Thus, utilization of these dipeptides required one of these two systems.

In the case of the dipeptides Ala-Tyr and Tyr-Ala, utilization was mediated by Ptr2p. Growth on these dipeptides was abolished by deletion of *PTR2* alone, whereas deletion of *DAL5* alone had no apparent effect. In contrast, the utilization of Ala-Leu involved both Ptr2p and Dal5p, but the relative contributions of these two activities varied between strains. Similar results were obtained using both solid (Figure 4) and liquid media (2 mM Ala-Leu; Figure S1). Ala-Leu could be utilized in strains RM8 and YAT7 via either Ptr2p or Dal5p, but Ptr2p was necessary and sufficient for wild-type growth levels. Strains W303 and Y55 did not exhibit this functional overlap in Ala-Leu utilization. W303-*ptr2*Δ grew as well as wild-type W303 on Ala-Leu, but W303-*dal5*Δ was completely unable to grow. In Y55, the same deletions created the opposite effect: Y55-*ptr2*Δ could not grow, whereas Y55-*dal5*Δ grew as well as wild-type Y55. Thus, Y55 relied upon Ptr2p for utilization of the assayed dipeptides, whereas W303 relied predominantly on Dal5p.

### The Scope of the Contribution of Dal5p to Di/Tripeptide Utilization

**Figure 5:**
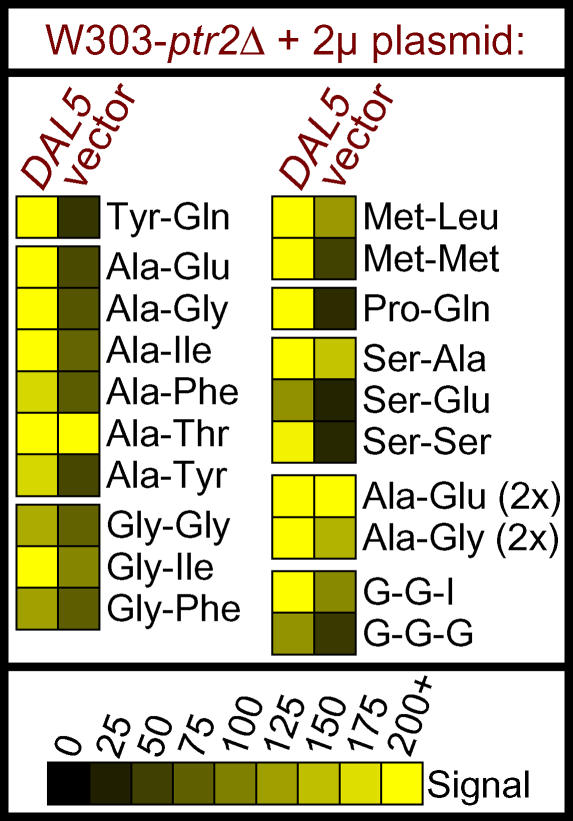
Utilization of a Subset of Dipeptides Is Enhanced in W303-*ptr2*Δ upon Overexpression of *DAL5* Strain W303-*ptr2*Δ was transformed with either the vector pRS426 or the plasmid pRS426-*DAL5*, a 2-μm plasmid containing a genomic fragment including *DAL5,* and phenotyped using the PM assay. The nitrogen sources shown comprise all instances in which the signal values met our reliability criteria (see Figure 2) and the presence of pRS426-*DAL5* altered the signal value by more than 50 units relative to the vector control. Dipeptides followed by “(2×)” are present in the PM assay plates at twice the concentration of the other dipeptides.

Next, we used the PM assay to determine how many di/tripeptides could be utilized as nitrogen sources through the activity of the *DAL5* gene. The gene was subcloned into a clean 2-μm vector, from the original plasmid that was isolated from the genomic screen, and tested in strain W303-*ptr2*Δ. Unlike the effect of overexpressing *PTR2* (in the *cup9*Δ strain), the multi-copy *DAL5* plasmid only enhanced growth on a small number of di/tripeptides (Figure 5). With the exception of Tyr-Gln, these di/tripeptides were all non–N-end rule dipeptides. N-terminal alanine, glycine, and serine residues were particularly favorable to Dal5p-mediated growth.

To determine whether Dal5p activity was redundant or supplemental to that of Ptr2p, the specific contribution of Dal5p to di/tripeptide utilization was explored using PM assays of the strains Y55-*dal5*Δ and RM8-*dal5*Δ (full data presented in Figure S2). In the case of Y55, deletion of *DAL5* had negligible impact upon dipeptide utilization (Figure S2B and Figure 4). Because the strongest growth defect in wild-type Y55 was in the utilization of those non–N-end rule dipeptides that we have found to depend on Dal5p, we speculated that Y55 might lack Dal5p activity. This hypothesis was verified by a PM assay of Y55-*ptr2*Δ. Unlike our earlier assays of *ptr2*Δ mutants in YAT7, RM8, and W303, deletion of *PTR2* in Y55 completely eliminated growth on all dipeptides and tripeptides (Figure S2). Thus, Y55 relies solely upon Ptr2p for the transport of dipeptides.

**Figure 6:**
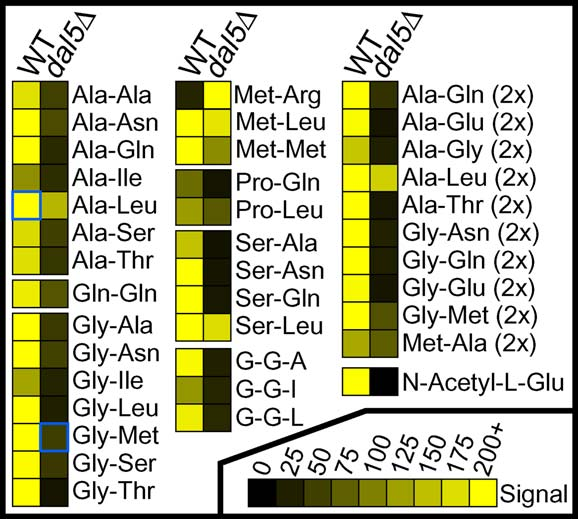
Deletion of *DAL5* in Strain RM8 Strongly Affects the Utilization of a Subset of Assayed Dipeptides All instances in which RM8 and RM8-*dal5*Δ signal values differ by more than 50 units and conform to our reliability criteria (see Figure 2) are presented. Dipeptides followed by “(2×)” are present in the PM assay plates at twice the concentration of the other dipeptides.

In contrast, Dal5p contributed substantially to the utilization of dipeptides in RM8. All of the dipeptides that supported more robust growth in the presence of Dal5p belonged to the non–N-end rule category, and the majority contained the small N-terminal residues alanine or glycine (Figure 6 and data not shown). Deletion of *DAL5* in strains RM8 and W303 did not significantly affect the utilization of any non-peptide nitrogen sources, except that growth was eliminated on N-acetyl-L-glutamate, an intermediate in arginine biosynthesis [41] (Figure S2C). (The fact that N-acetyl-L-glutamate utilization is an indicator of Dal5p activity explains why wild-type Y55 cannot utilize this substrate; see Figure 2E.) Clearly, *DAL5* makes an important contribution to dipeptide utilization in yeast, and variations in *DAL5* activity contribute to the naturally occurring variation in nitrogen utilization in diverse strains.

### Elimination of Ptr2p and Dal5p Uncovers Additional Dipeptide-Utilization Activities

To determine whether Ptr2p and Dal5p account for all dipeptide utilization in the PM assays, we analyzed *ptr2*Δ*dal5*Δ double mutants in two strains: W303, which showed the greatest dependence on Ptr2p-independent mechanisms, and RM8, which relied heavily upon both Ptr2p and Dal5p (Figure S2). In both strains, deletion of these two genes abolished all growth on most tested dipeptides and tripeptides in both strains. The most notable exception was that W303-*ptr2*Δ*dal5*Δ, but not RM8-*ptr2*Δ*dal5*Δ, retained the capacity to utilize all dipeptides with C-terminal asparagine residues (Figure S2; boxes with red borders). The obvious candidate for the underlying molecular determinant of this activity is the periplasmic asparaginase II (see Discussion). Since this protein is encoded by four copies of the *ASP3* gene interspersed among rRNA and Ty loci in the genome, the origin of the phenotype was not amenable to genetic analysis.

A second strong exception to the requirement for Ptr2p and Dal5 was the utilization of Met-Arg by RM8, which was actually enhanced by the *dal5*Δ mutation (Figure 6) and remained robust in the *ptr2*Δ*dal5*Δ double mutant (Figure S2). Similar analyses of YAT7, W303, and Y55 mutants (Figure S2B and data not shown) indicated that the ability to robustly utilize Met-Arg in the absence of Ptr2p and Dal5p function was unique to RM8. Closer examination of the PM assay plates revealed that this Ptr2p- and Dal5p-independent activity might affect the utilization of other dipeptides as well. When PM assay plates containing RM8-*ptr2*Δ*dal5*Δ were incubated at 30 °C for an additional 1 to 3 d following the PM assay, growth was seen in wells containing other dipeptides—specifically, those with C-terminal arginine residues (data not shown). This delayed-growth phenotype did not require the function of Dal5p or Ptr2p and was not observed in plates containing either W303-*ptr2*Δ*dal5*Δ or Y55-*ptr2*Δ. Thus, it represented a fourth distinct dipeptide utilization activity.

### Inter-Strain Variation in Sensitivity to the Toxic Dipeptide Ala-Eth

**Figure 7:**
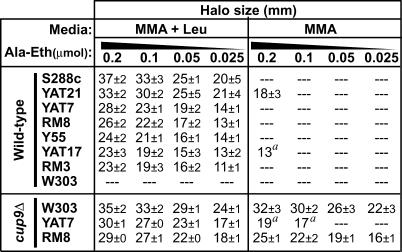
Halo Assay for Uptake of the Toxic Ala-Eth Dipeptide Cells were plated onto MMA with or without 30 μg/ml leucine, an inducer of *PTR2* expression. Halo diameters in the presence of discs containing the indicated Ala-Eth concentrations are presented as the mean ± standard deviation. (*ptr2*Δ strains were also tested, and exhibited no halos.) ^a^Halo boundary was indistinct, often slowly receding as cells closer to the disc began to grow.

The benefit of efficient utilization of di/tripeptides as a nutrient source is likely to be counterbalanced by the vulnerability associated with indiscriminate peptide transport. To assay inter-strain variation in vulnerability to toxic peptides, the panel of diverse *S. cerevisiae* strains was exposed to a dipeptide containing L-ethionine (Eth), a toxic methionine analogue (Figure 7). The purine derivative allantoin was provided as a nitrogen source [42] to eliminate the nutritional requirement for dipeptide uptake. Discs containing different concentrations of Ala-Eth were applied to lawns of cells, creating gradients of dipeptide concentration. Thus, the diameter of the halo of growth inhibition surrounding a disc is an indicator of dipeptide import.

In the standard halo assay, minimal medium with allantoin (MMA) is supplemented with 30 μg/ml leucine to induce *PTR2* expression (MMA+Leu medium). Under these conditions, wild-type strains varied substantially in sensitivity to Ala-Eth toxicity (Figure 7). S288c was the most sensitive to the dipeptide, whereas W303 was completely resistant. In the absence of the leucine inducer, only the clinical isolates YAT17 and YAT21 were sensitive to Ala-Eth (Figure 7).

Deletion of *PTR2* alone was sufficient to confer complete resistance to the toxic dipeptide under the conditions tested (RM8-*ptr2*Δ and YAT7-*ptr2*Δ were tested; data not shown). While this might suggest that Ptr2p is the primary transporter of Ala-Eth, we note that Dal5p may also transport allantoin [43,44], raising the possibility that allantoin competed with Ala-Eth for Dal5p-mediated uptake. Indeed, Ala-Met, which is structurally similar to Ala-Eth, supports growth in *ptr2*Δ strains (see Figure 3B) and *dal5*Δ strains (Figure S2B), suggesting that Dal5p and Ptr2p both contribute to the vulnerability of yeast cells to Ala-Eth.

**Figure 8:**
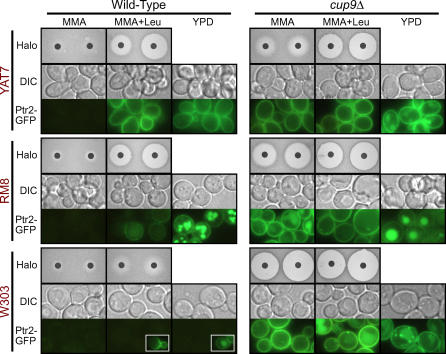
Inter-Strain Variation in *PTR2* Expression and Sensitivity to the Toxic Dipeptide Ala-Eth Media composition and the absence of Cup9p affect expression of GFP-tagged *PTR2* and Ala-Eth toxicity. Three types of media were tested: MMA, MMA+Leu, and YPD. “MMA” refers to minimal nitrogen medium containing 1 mg/ml allantoin as the nitrogen source. “+Leu” refers to the addition of 30 μg/ml leucine, an inducer of *PTR2* expression. YPD is a standard rich *S. cerevisiae* medium; the presence of methionine precludes its use for the halo assay. The left and right discs in each halo assay panel contained 0.1 μmoles and 0.2 μmoles of Ala-Eth, respectively. Microscopy was conducted using cells grown to log phase in the indicated media. Three strains were imaged: W303, RM8, and YAT7. In each strain, the endogenous *PTR2* gene contained a C-terminal tag consisting of a FLAG epitope, two copies of GFP, and a 6xHis tag. The insets depict representative cells from the small subpopulation of W303 cells that exhibited fluorescence.

Elimination of the *PTR2* repressor Cup9p enhanced Ala-Eth sensitivity in all strains tested, yet it also revealed additional diversity among the strains (Figure 7, *cup9*Δ). The resistance of strain W303 to Ala-Eth was overcome by the *cup9*Δ mutation to the extent that the W303-*cup9*Δ mutant exhibited higher sensitivity to Ala-Eth than *cup9*Δ mutants of either RM8 or YAT7. All of the *cup9*Δ mutant strains exhibited some sensitivity to Ala-Eth in the absence of the leucine inducer. Curiously, despite the robust dipeptide import characteristic of the YAT7 strain, YAT7-*cup9*Δ produced much smaller halos under these conditions than either W303-*cup9*Δ or RM8-*cup9*Δ (Figure 7; see also Figure 8). Some of the inter-strain variation observed in the halo assay might reflect varying sensitivity to ethionine. However, since both changes in media composition and the *cup9*Δ mutations affected strains differently, this diversity is clearly shaped by the complex regulatory controls governing dipeptide import.

### Expression and Localization of Ptr2p Varies Substantially among Strains

Strain W303 was both highly resistant to the toxic Ala-Eth dipeptide and limited in dipeptide utilization capacity. Therefore, we asked whether *PTR2* expression in this strain differed from that of the other strains tested. To directly visualize the expression of *PTR2,* a C-terminal FLAG-2xGFP-6xHis tag was added to the endogenous *PTR2* gene of strains W303, RM8, and YAT7 by site-specific homologous recombination. These modified strains retained a similar capacity to utilize dipeptides (data not shown). To facilitate comparison of *PTR2* expression with the results of the halo assay, expression of green fluorescent protein (GFP)-tagged *PTR2* was assayed in a liquid version of the MMA and MMA+Leu media, and fluorescence was measured using a uniform exposure level (Figure 8; halo assay images included for comparison).

The presence of Ptr2p at the plasma membrane correlated well with the extent of Ala-Eth toxicity observed in the halo assay (except that fluorescence was weaker in RM8 than might have been expected from its strong Ala-Eth sensitivity in MMA+Leu). In all other cases, conditions promoting strong Ala-Eth sensitivity also induced strong *PTR2* expression. Strikingly, wild-type W303, which was resistant to Ala-Eth, exhibited virtually no Ptr2p fluorescence. The *cup9*Δ mutation restored strong *PTR2* expression and also restored Ala-Eth sensitivity. Thus, the resistance of W303 to Ala-Eth in the halo assay resulted from an absence of Ptr2p at the plasma membrane. Moreover, these results confirm that W303 contains a functional *PTR2* gene subject to atypical regulation.

### Additional Aspects of *PTR2* Regulation

Retrograde recycling of membrane-bound Ptr2p is thought to represent an additional layer of regulatory control of dipeptide import (H. Cai and J. Becker, unpublished data). Although GFP-tagged Ptr2p was typically localized to the membrane, when strain RM8 was grown in YPD, additional fluorescent signal was also observed in the vacuole and in what appeared to be large vesicles. The high levels of *PTR2* expression in the rich YPD media was itself surprising, since *PTR2* is thought to be subject to nitrogen catabolite repression in the presence of preferred nitrogen sources [28]. Although YAT7 also exhibited robust *PTR2* expression under these conditions, Ptr2p was confined to the plasma membrane. This inter-strain variation was likely influenced by cellular Ptr2p levels, as *cup9*Δ mutants of RM8, YAT7, and W303 all exhibited robust *PTR2* expression and some intra-cellular Ptr2p localization in YPD media (Figure 8). However, although wild-type RM8 and YAT7 both exhibited strong Ptr2p fluorescence in YPD, only RM8 exhibited abundant intra-cellular compartmentalization of Ptr2p, indicating that retrograde transport of Ptr2p is yet another regulatory process that varies between strains in *S. cerevisiae*.

The regulation of *PTR2* in W303 differed significantly from the other strains analyzed in this study. Considering the possibility that polymorphisms within the *PTR2* promoter region may account for the limited *PTR2* expression in W303, we sequenced an 889–base pair (bp) region immediately upstream of the *PTR2* start codon in the strains W303, S288c, RM8, and YAT7 (Table S1). The promoter region in W303 differed from that of S288c at only one base, a C→T transition at position −799. However, this polymorphism was shared by the strain RM8, which unlike W303 is sensitive to Ala-Eth and exhibits robust *PTR2*-dependent utilization of N-end rule dipeptides. Thus, differences in promoter sequence are not a likely source for the altered regulation of *PTR2* in W303.

Telomeric regions are common sites of epigenetic regulation, and *PTR2* is located in proximity (~50 kb) to a telomere. In our analyses of GFP-tagged *PTR2,* a small subpopulation (1 in 50 to 1 in 1,000, depending on conditions) of strongly fluorescent W303 cells was consistently observed during growth in either the MMA+Leu medium or in YPD (Figure 8; inset). W303 cells exhibiting robust *PTR2* expression also arose spontaneously at a high frequency (>0.1%) on solid media. These cells maintain competency for *PTR2* expression following serial propagation on media that relieve selection for active dipeptide transport (J. Manjrekar and S. Lindquist, personal communication). These switching events were far too frequent to reflect mutation, suggesting that an epigenetic switch exists that can stably confer competency for PTR2 expression in W303 cells.

## Discussion

Our results establish that the rich inter-strain diversity and genetic tractability of *S. cerevisiae* can be combined with high-throughput phenotyping to provide new insights into basic cellular processes. Using this approach, we have uncovered previously unrecognized functions and regulatory systems governing the utilization of dipeptides as a nitrogen source. The natural diversity in these systems included variation in sensitivity to a toxic dipeptide, suggesting that dipeptide utilization can be considered in the context of an adaptive landscape shaped by opposing selective pressures.

### Using High-Throughput Phenotyping to Study Di/Tripeptide Utilization

The exploration of natural diversity using high-throughput phenotyping techniques has the potential for tremendous synergy with the existing high-throughput technologies that have shaped our understanding of the cellular circuitry of *S. cerevisiae*. The pursuit of an integrated understanding of yeast functional genomics and proteomics (reviewed in [45]) has been enabled by numerous powerful high-throughput approaches. For example, genetic networks have been elucidated by high-throughput screens for synthetic lethality [46], protein interaction networks have been mapped using mass spectrometry [47,48], and transcriptional networks have been elucidated by the now-ubiquitous technique of transcriptional profiling using microarrays and by the mapping of physical interactions between transcription factors and promoters [49]. The technology for global analysis of growth phenotypes remains largely underdeveloped and unexploited—despite the fact that it is phenotype that determines how cellular systems interface with selective pressures. Thus, the broad synthesis of our understanding of genomic, transcriptional, and phenotypic diversity is contingent upon the development of robust technologies for high-throughput phenotyping.

Here, we have tested one of the first such systems by assaying dipeptide and tripeptide utilization on a previously unfeasible scale. The PM phenotypic assay allows simultaneous testing of approximately 2,000 growth conditions, providing a tremendous savings in time and labor. The technology was typically very robust and provided reliable and reproducible data that were easily manipulated using the accompanying software. However, the PM technology also has several drawbacks that need to be addressed before this technology is broadly suitable for use by the yeast research community. Due to the initial design focus on generalized bacterial phenotyping, many of the available PM assay plates are not optimized for *S. cerevisiae*. Thus, the number of useful growth conditions is considerably less than 2,000. This issue is compounded by the high cost of the PM assay plates. Further, different batches of media and PM assay plates provided by the manufacturer often affected reproducibility. Such technical difficulties are not atypical for a new technology, and the power of such tools in exploring the phenotypic diversity of *S. cerevisiae* is clear. Hopefully, the importance of high-throughput phenotyping will lead to reduced costs and enhanced suitability of these technologies.

### Uncovering Unexpected Complexity in Di/Tripeptide Utilization

Through extensive phenotyping, we found that Ptr2p is but one component of a multifaceted system in *S. cerevisiae* governing the utilization of dipeptides as nitrogen sources. The discovery of these additional components was made possible by the dramatic inter-strain variation observed in the contributions of these activities to dipeptide utilization (summarized in Table 2). We have clearly identified one previously unknown component of this system—the allantoate/ureidosuccinate permease Dal5p—and have defined parameters of two others that suggest mechanistic explanations.

**Table 2:**
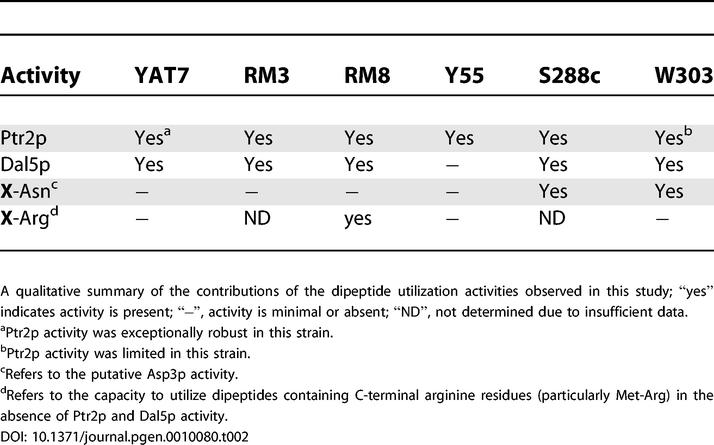
Activities Contributing to the Utilization of Dipeptides as a Nitrogen Source in Each Strain Background

Of these four components, Ptr2p exhibits the broadest dipeptide utilization activity. Relief of *PTR2* repression by deletion of *CUP9* facilitated robust growth on nearly all di/tripeptides tested. This was even true of W303, which otherwise grew on only a limited subset of the N-end rule dipeptides that supported growth of the other wild-type strains. This restricted utilization of potential Ptr2p substrates provides a window into the complex *PTR2* regulatory system. For example, one can ask why W303 is capable of growing on Trp-Arg, but not Arg-Trp (see Figure 3A), even though both are N-end rule peptides composed of identical residues. One possibility is that the N-end rule pathway in W303 is more responsive to Type 1 ligands (basic N-terminal residues; e.g., Arg) than Type 2 substrates (bulky hydrophobic N-terminal residues; e.g., Trp). Alternatively, substrate preference may arise from dipeptidase specificity [50] resulting in more robust hydrolysis of Trp-Arg upon import. Although beyond the scope of this analysis, additional insights and hypotheses can be derived from the wealth of data provided by our high-throughput analysis, and the approach developed here—the application of a diverse panel of strains to the PM assay to analyze di/tripeptide utilization—can be employed in future studies to enrich our understanding of *PTR2* regulation and Ptr2p function.

Dal5p, a second component of the dipeptide utilization system, was previously identified as a permease for the pyrimidine biosynthetic precursor ureidosuccinate [51] and the nitrogen source allantoate [43]. Expression of *DAL5* is subject to complex regulatory control by the nitrogen catabolite repression system [43,52,53]. It is therefore fitting that Dal5p also is involved in the utilization of dipeptides as a nitrogen source. With the exception of Tyr-Gln, all Dal5p di/tripeptide substrates belonged to the non–N-end rule class. Many of the dipeptides supporting growth in RM8-*ptr2*Δ (see Figure 3) did not support growth in RM8-*dal5*Δ (see Figure 6). Thus, Dal5p-mediated dipeptide transport is not redundant to that of Ptr2p, but rather a complementary activity that enhances the range of dipeptides that can be utilized.

A third component, evident in strains W303 and S288c, governs the utilization of dipeptides containing C-terminal asparagine residues as nitrogen sources (see Figure 2). Several lines of evidence support the conclusion that the Asp3p periplasmic asparaginase facilitates the utilization of these substrates. Asp3p activity is closely linked to nitrogen utilization. Nitrogen starvation has been demonstrated to up-regulate *ASP3* expression and promote the secretion of asparaginase II [54]. Furthermore, the enzymatic activity of asparaginase II hydrolyzes either L- or D-asparagine into ammonia and aspartate, facilitating the use of D-asparagine as a nitrogen source [55,56]. Of the strains tested, only W303 and the related laboratory strain S288c were able to utilize D-asparagine in the PM assay (see Figure 2E). Genomic analyses utilizing microarrays have noted a lack of detectable *ASP3* in Y55 [57] and a clinical isolate, YJM789 [3]. Neither Y55 nor our clinical isolate, YAT7, were capable of utilizing the putative Asp3p substrates (see Figure 2). Most important, the asparaginase of the bacterial plant pathogen *Erwinia carotovora* has been shown to deamidate C-terminal, but not N-terminal, asparagine residues of small peptides [58]. This specificity precisely matches the dipeptide utilization phenotypes observed for W303 and S288c. Both strains are unable to utilize the two dipeptides with N-terminal asparagine residues present in the PM nitrogen plates (see Figure 2B), but are able to utilize all dipeptides containing C-terminal asparagine residues (see Figure 2; boxes with red borders). To our knowledge, our study provides the first evidence for such an activity in *S. cerevisiae,* in which mechanisms of extracellular peptide processing are rare.

A fourth component of the dipeptide utilization system, evident in strain RM8 but not W303, governs the use of dipeptides containing C-terminal arginine residues, and Met-Arg in particular. Utilization of Met-Arg is clearly enhanced by deletion of *DAL5* (see Figure 6) and persists in the *ptr2*Δ*dal5*Δ double mutant (Figure S2), and thus represents another previously unrecognized mechanism of dipeptide utilization. Given the specificity for a nitrogen-rich C-terminal residue, an arginase activity comparable to the asparaginase activity of Asp3p would seem a likely candidate.

### Balancing the Benefits and Potential Risks of Robust Di/Tripeptide Import

Variation in the relative strengths of the activities contributing to dipeptide import can strongly impact the range of dipeptide substrates utilized by the cell. Although di/tripeptides can serve as a valuable source of nutrients, evolutionary pressures opposing indiscriminate import may reinforce mechanisms promoting transport specificity. One such pressure might be the need to avoid import of antifungal agents that exploit the di/tripeptide transport machinery to gain entry into the cell (reviewed in [22]).

Of the di/tripeptide-utilization activities characterized in this study, the Ptr2p transporter provides the means to transport the broadest spectrum of dipeptide substrates (see Figure 3), yet it is also the source of vulnerability to the toxic Ala-Eth dipeptide (see Figure 7). Thus, strains that exhibit robust *PTR2* expression, such as the clinical isolate YAT7, efficiently utilize a broad spectrum of dipeptides as a nitrogen source (see Figure 2), but are also vulnerable to Ala-Eth toxicity (see Figure 7). In contrast, the limited *PTR2* expression in strain W303 provides protection from Ala-Eth at the cost of a more limited capacity to utilize di/tripeptides. However, in certain growth conditions, a fraction of W303 cells exhibit robust *PTR2* expression, suggesting a possible epigenetic mechanism for employing both high- and low-risk di/tripeptide utilization strategies in an isogenic population.

The optimal strategy for dipeptide utilization will vary between environments. For instance, in the presence of toxic peptidomimetics that resemble Dal5p substrates, strain Y55 would have a significant fitness advantage over the other strains tested in this study, as it exhibits no detectable Dal5p activity (Figure S2). In environments with a strong risk of exposure to a variety of toxic di/tripeptides, the risks of Ptr2p- and Dal5p-mediated import may outweigh the potential nutritional benefits. In such cases, the utilization of a limited subset of di/tripeptides may represent a low-risk mechanism by which some nutritional benefit can be extracted from the available di/tripeptides. For example, extracellular release of ammonia from C-terminal asparagine residues by Asp3p would serve as a more limited, but risk-free, means of obtaining nitrogen from dipeptides. In contrast, in an environment in which nitrogen sources are scarce and the risk of importing toxic species is minimal, robust expression of *PTR2* would be a beneficial trait. We note that the clinical isolates YAT7 (see Figure 2), YAT17, and YAT21 (data not shown) exhibit the most robust dipeptide utilization of the strains studied, perhaps reflecting the environmental pressures of the strains' atypical growth environment in the human host. Further investigation into the ecology, population structure, and population genetics of *S. cerevisiae* would benefit future attempts to link phenotypic diversity to likely environmental pressures.

High-throughput phenotyping and genetic diversity can provide new insights into the genetic determinants governing adaptation to the nutritional and chemical challenges of diverse environments. Our study represents a first-case example of how this approach can be utilized to elucidate metabolic processes and provide a broader evolutionary context within which the observed natural diversity can be considered.

## Materials and Methods

### Media and reagents.

Synthetic defined (SD), YPD, and 5-FOA (BioVectra) media were made using standard formulations [59]. When needed, G418 (Gibco, Carlsbad, California, United States) was added to the YPD medium at 200 μg/ml to select for Kan^r^. To select for HygB^r^, Hygromycin B (American Bioanalytical, Natick, Massachusetts, United States) was added to YPD at 250 μg/ml. All dipeptides used outside of the PM assays were obtained from Bachem California (Torrance, California, United States). A minimal medium (MM), supplemented with the indicated nitrogen sources, was utilized for the Bioscreen C growth curve assays (Growth Curves USA, Piscataway, New Jersey, United States). MM contained 2% glucose, 0.17% yeast nitrogen base without (NH_4_)_2_SO_4_ and amino acids (Qbiogene, Irvine, California, United States), 18 mg/L uracil (omitted for *URA3* plasmid-bearing strains), and 2% agar (for solid media). The MMA used in the halo and GFP assays was identical to MM, except that it contained 1 g/l allantoin as a nitrogen source and a slightly different concentration of uracil (20 mg/l). The Biolog PM media contained 50 mM glucose, 1 mM disodium pyrophosphate, 2 mM sodium sulfate, and a proprietary tetrazolium dye mix and IFY-0 medium. IFY-0 is a basal medium, lacking nitrogen, carbon, phosphate, and sulfur sources, which are supplemented as needed.

### Plasmid construction.

Construction of the pMS2 plasmid is described in [60]. The plasmid pPTR2-2xGFP-SFH-KanR was constructed by addition of tandem copies of GFP (2xGFP) and a *kan^r^* selectable marker into plasmid pMS2. First, primers PTR2-FLAG-GFP-F and PTR2-FLAG-GFP-R (all primer sequences are supplied in Table S2) were used to amplify a 1.5-kb fragment containing 2xGFP from plasmid pKW430 (a gift of Dr. Mary Miller, Rhodes University [61]) and an additional 40 bp of flanking sequence homologous to pMS2. The plasmid pMS2 was then linearized at the unique restriction site AgeI, located between the FLAG and His tags. 2xGFP was then inserted between these two tags by homologous recombination, creating pMS4. A HincII fragment containing *kanMX4* from pFA6a-kanMX4 [62] was then inserted into the NaeI site of pMS4, which lies beyond the stop codon following the 6xHis tag, creating the final pPTR2-2xGFP-SFH-KanR plasmid.

The plasmid pRS426-DAL5 was created by cloning an EcoRV genomic fragment from a plasmid recovered in the genomic screen (see below) into the 2-μm vector pRS426 (ATCC#77107; [63]). This fragment spans a region from −1334 to +2908, relative to the start codon of the *DAL5* gene.

pUG6-HygB was created by replacement of the *kanMX* marker in pUG6 [64] with a *hphMX* cassette from pAG32 [65]. First, a PCR fragment containing the *hphMX* cassette was amplified from pAG32 using primers O-199 and O-200 and digested with MluI and BsmI. The resulting fragment was then inserted into the similarly digested pUG6.

### Strain construction.

The source and origins of the strains used in this study are indicated in Table 1. To make all strains MATa *ura3,* some modifications were made to eliminate amino acid auxotrophies, mutate *URA3,* and change mating types (described in detail in Protocol S1). All transformations were conducted using the standard lithium acetate technique [66].

*PTR2* was deleted with a short-flanking homology (SFH) cassette amplified from plasmid pFA6a-GFP(S65T)-kanMX6 [62] (pAG32 [65] in the case of Y55; the GFP portion of the plasmid was not amplified). This deletion cassette replaced the *PTR2* region spanning +77 to +1781, relative to the start codon, with a *kanMX* marker (*hphMX* in the case of Y55). *CUP9* was deleted with a SFH cassette amplified from plasmid pFA6a-GFP(S65T)-kanMX6, removing the *CUP9* region spanning +41 to +878, relative to the start codon. *DAL5* was deleted with a *hphMX* marker from a SFH cassette amplified from pAG32. This deletion cassette eliminated the *DAL5* region spanning −70 to +1594, relative to the start codon.

Addition of a FLAG-2xGFP-6xHis tag to the C-terminus of endogenous *PTR2* was accomplished with a SFH cassette amplified from plasmid pPTR2-2xGFP-SFH-KanR. Proper integration was ensured by selection on G418 media and verification by colony PCR. Final confirmation of proper integration was obtained by observation of membrane-localized GFP fluorescence in MMA+Leu medium.

### PM assays of nitrogen source utilization.

A full list of the nitrogen sources tested in the PM assay plates is provided in Dataset S4. Each well of the PM plates contains a very low micromolar amount of amino acids, at a level insufficient to complement auxotrophies or serve as an adequate nitrogen source. All dipeptides are present at the same concentration (between 1–4 mM, the precise concentration is proprietary information), with the exception of the small set of dipeptides present in PM03, which are present at double the concentration of the others. (These are notated as (2x) when presented in the figures.)

Strains were prepared for inoculation into the PM assay plates as follows. Glycerol stocks were streaked to YPD plates and incubated at 30 °C for approximately 48 h. Next, two to three colonies from each strain were restreaked to one section of a fresh YPD plate and incubated overnight at 30 °C. In the morning, cells on each YPD plate were spread over the plate to form a thin layer and incubated an additional 4–6 h to ensure that most cells were actively growing. This technique also served to avoid the late-growth clumping encountered with some wild strains. In the case of strains containing plasmids, standard SD–ura dropout plates were used in place of YPD.

Cells were then inoculated into sterile water in 20 × 100 mm test tubes and adjusted to a transmittance of 63% (~5×10^6^ cells/ml). This 48× concentration cell suspension also contained 14.4 mM uracil, except when plasmids were used. The cell suspension was then reduced to 1× concentration by dilution into the Biolog PM media; 100 μl of this mix was then inoculated into each well of the PM assay plates PM03, PM06, PM07, and PM08. The plates were then sealed with sterile Axygen SealPlate film (Union City, California, United States), placed in the OmniLog reader (Biolog), and incubated for 48 h at 30 °C. The OmniLog reader photographs the plates at 15-min intervals, converting the pixel density in each well to a signal value reflecting cell growth and dye conversion.

After completion of the run, the signal data were compiled and analyzed. Signal data were exported from the Biolog software, compiled using Microsoft Excel (Redmond, Washington, United States), and then visually represented using Java TreeView v1.0.8 [67] and Adobe Illustrator (San Jose, California, United States). We chose to export the average and maximum signal value for each well and then average the two values. In doing so, we were able to represent the full time course by a single number and weight the value towards latter time points. Shadows cast over perimeter wells in the OmniLog machine sometimes raised the basal signal level of these wells. This was accounted for by determining a “baseline” signal level for each well by calculating the average signal level for the first 2 h of the run. This value was then subtracted from the previously calculated signal value, yielding a final signal value. These calculations can be summarized as follows:


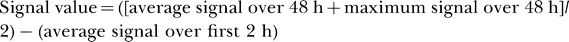


In all cases, a minimum of two replicate PM assay runs were conducted, and the average of the signal values was used. Additionally, when non–wild-type strains were assayed, two independently constructed deletion strains were always tested. The PM assay data used to calculate the signal values presented in this study are provided in Dataset S4. Images of all signal curves are provided in Datasets S1–S3.

To ensure that dye reduction was not occurring in the absence of growth, all PM plates were carefully examined following each run. The plates were inverted upon a light-box such that cells were visible in wells that supported growth. This approach revealed that dipeptides containing lysine, histidine, and cysteine residues, as well as the amino acid histidine, sometimes supported reduction of the dye in the absence of growth. (Dipeptides containing these residues were omitted from the analysis; see Results.) Two additional control measures were taken to ensure that the correlation between dye reduction and cell density was robust. First, the PM assay was conducted once with each wild-type strain in the absence of the tetrazolium dye. Second, several PM runs were plated as dilution series to YPD media at the conclusion of the 48-h run. These additional measures confirmed that, with the exceptions noted above, the PM assay signal is a reliable indicator of growth in the presence of the nitrogen sources discussed in this study.

### Bioscreen growth curve analysis.

A selection of PM assay dipeptide-utilization phenotypes were verified using a Bioscreen C (Growth Curves USA) plate reader. Strains were cultured as described for the PM assay, but were inoculated at an optical density at 595 nm (OD_595_) of 0.02 into MM supplemented with the indicated nitrogen source. The mixture was inoculated into a 100-well plate and incubated in the Bioscreen C at 30 °C with heavy shaking. Measurements of OD_600_ were made at 45-min intervals. Each growth curve was conducted in duplicate wells and was repeated at least once.

### Screen for Ptr2p-independent dipeptide utilization.

The screen was conducted using a pRS202-based genomic library (2 μm; URA3) containing Sau3AI-digested genomic DNA with an average insert size of 6–8 kb (gift of Dr. Gerald Fink; originally constructed by Connelly and Hieter from a strain congenic with S288c [68]). A full description of the screen can be found in Protocol S2.

### Halo assay of Ala-Eth toxicity.

The sensitivity of deletion mutants to the toxic dipeptide Ala-Eth was measured as previously described [29]. Ethionine (Eth) is an analog of methionine, and utilization of Eth causes cell death. The tested strains were grown overnight in MMA. Yeast cells were harvested and washed three times with sterile, distilled water, then counted and adjusted to 5 × 10^6^ cells/ml. One ml of the cell suspension was added to 0.8% noble agar (3 ml) and plated onto MMA with or without leucine (30 μg/ml), an inducer of *PTR2* expression. Four 6-mm sterile paper disks containing either 0.2, 0.1, 0.05, or 0.025 μmoles of Ala-Eth were placed on the lawn of cells. The size of the halo was measured after two days of incubation at 30 °C. Between two and four replicates were conducted for each assay. Although strains Y55 and RM3 were unable to utilize allantoin in the PM assay (see Figure 2E) and in additional Bioscreen C assays using liquid medium (0.5 mM allantoin; data not shown), both strains were capable of utilizing allantoin in the solid halo assay medium.

### Sequencing upstream of *PTR2.*

Although the region comprising the *PTR2* promoter has yet to be clearly defined, the sequenced span, an 889-bp region immediately upstream of the *PTR2* start codon, should contain the key regulatory elements. The region bound by the Cup9p repressor has been narrowed to the region spanning −897 to −448 [34], relative to the *PTR2* start codon. In addition, a predicted amino acid–dependent upstream activator sequence (UAS_AA_) is located between −777 and −760 [69]. Genomic DNA was prepared using the “smash and grab” technique [70]. A 927-bp fragment containing the desired sequence was amplified with primers O-321 and O-324, using an equal amount of Taq and Vent polymerases. Each reaction mix was split into eight aliquots and repooled following amplification. This approach was taken to help control for the possibility that a single replication error could propagate through the entire sample. PCR products were then purified with a Qiagen PCR purification kit (Valencia, California, United States) and sequenced in both directions by Northwoods DNA Inc. (Solway, Minnesota, United States) using oligos O-321, O-322, O-323, and O-324. Sequencing data were analyzed using Sequencher DNA sequencing software (Gene Codes Corporation, Ann Arbor, Michigan, United States).

### GFP microscopy.

To test the localization of GFP-tagged Ptr2p, yeast strains carrying *PTR2* with a C-terminal FLAG-2xGFP-6xHis tag were grown at 30 °C in MMA, MMA+Leu, or YPD media. Yeast cells were collected during log-phase and imaged. The GFP signal was observed by fluorescence microscopy with a 470–490-nm excitation wavelength and 515-nm emission filter fitted to an Olympus microscope (Melville, New York, United States). Both fluorescent and differential interference contrast (DIC) images were taken with a MicroFire camera (Model S99809; Olympus).

## Supporting Information

Dataset S1 PM Signal Curves for Data Presented in Figure 2
Each image provides the signal curves generated by the OmniLog software in each well of the four 96-well plates that constitute the PM nitrogen source utilization assay (see Dataset S4 for full listing of plate contents). The *x-*axis of each signal curve represents the 48-h time course. The *y-*axis represents the signal intensity, an indirect measure of growth. The different colored lines used for the signal curves represent independent replicates of the PM assay.
(2.1 MB DOC)

Dataset S2 PM Signal Curves for Data Presented in Figure 3
See Dataset S1 legend for details.
(2.2 MB DOC)

Dataset S3 PM Signal Curves for Data Presented in Figure S2
See Dataset S1 legend for details.
(3.1 MB DOC)

Dataset S4 Raw Quantitative PM Data Contains data used to calculate signal values and a complete listing of the contents of the PM plates utilized in this study.
(805 KB XLS)

Figure S1 Dal5p and Ptr2p Exhibit Strain-Dependent Contributions to the Utilization of Ala-Leu as a Nitrogen Source Wild-type, *dal5*Δ, *ptr2*Δ, and *ptr2*Δ*dal5*Δ versions of the indicated strains were grown at 30 °C in liquid MM containing 2 mM Ala-Leu as the sole nitrogen source. A few time points were omitted because of technical difficulties with the Bioscreen C growth curve machine.
(2.8 MB JPG)

Figure S2 Additional PM Signal Profiles Reveal the Varied Contribution of Dal5p to Di/tripeptide Import in Strains RM8, W303, and Y55 Refer to Figure 2 for details on data analysis and presentation. The column label *ptr2*Δ (vector) refers to strain W303-*ptr2*Δ transformed with the high-copy (2 μm) vector pRS426. The column label *ptr2*Δ (*DAL5*) refers to strain W303-*ptr2*Δ transformed with plasmid pRS426-DAL5. Note that the PM assays presented in this figure utilized a more recent manufacturing lot of the PM nitrogen plates than those presented in the other figures. Different lots can produce subtle differences in signal magnitude. The wild-type RM8 and Y55 PM data presented here were derived from the same lot to facilitate direct comparison.
(631 KB PDF)

Protocol S1 Construction of the Strains Used in This Study The source and origins of the strains used in this study are indicated in Table 1.
(39 KB DOC)

Protocol S2 Screen for Ptr2p-Independent Dipeptide Utilization (25 KB DOC)

Table S1 Polymorphisms in the Region Upstream of *PTR2,* Relative to the Standard S288c Sequence (28 KB DOC)

Table S2 Oligos Used in This Study (52 KB DOC)

### Accession Numbers

The Swiss-Prot (http://www.ebi.ac.uk/swissprot) accession numbers for the genes and gene products discussed in this paper are *ASP3* (P11163), Cup9p (P41817), *DAL5* (P15365), Ptr2p (P32901), and Ubr1p (P19812).

## Abbreviations

bp: base pair
GFP: green fluorescent protein
MM: minimal medium
MMA: minimal medium with allantoin
PM: Phenotype MicroArray
SFH: short-flanking homology

## References

[pgen-0010080-b001] Mortimer RK, Romano P, Suzzi G, Polsinelli M (1994). Genome renewal: a new phenomenon revealed from a genetic study of 43 strains of *Saccharomyces cerevisiae* derived from natural fermentation of grape musts. Yeast.

[pgen-0010080-b002] True HL, Lindquist SL (2000). A yeast prion provides a mechanism for genetic variation and phenotypic diversity. Nature.

[pgen-0010080-b003] Winzeler EA, Lee B, McCusker JH, Davis RW (1999). Whole genome genetic-typing in yeast using high-density oligonucleotide arrays. Parasitology.

[pgen-0010080-b004] Winzeler EA, Richards DR, Conway AR, Goldstein AL, Kalman S (1998). Direct allelic variation scanning of the yeast genome. Science.

[pgen-0010080-b005] Steinmetz LM, Sinha H, Richards DR, Spiegelman JI, Oefner PJ (2002). Dissecting the architecture of a quantitative trait locus in yeast. Nature.

[pgen-0010080-b006] Yvert G, Brem RB, Whittle J, Akey JM, Foss E (2003). Trans-acting regulatory variation in *Saccharomyces cerevisiae* and the role of transcription factors. Nat Genet.

[pgen-0010080-b007] Brem RB, Yvert G, Clinton R, Kruglyak L (2002). Genetic dissection of transcriptional regulation in budding yeast. Science.

[pgen-0010080-b008] Brem RB, Kruglyak L (2005). The landscape of genetic complexity across 5,700 gene expression traits in yeast. Proc Natl Acad Sci U S A.

[pgen-0010080-b009] Cavalieri D, Townsend JP, Hartl DL (2000). Manifold anomalies in gene expression in a vineyard isolate of Saccharomyces cerevisiae revealed by DNA microarray analysis. Proc Natl Acad Sci U S A.

[pgen-0010080-b010] Townsend JP, Cavalieri D, Hartl DL (2003). Population genetic variation in genome-wide gene expression. Mol Biol Evol.

[pgen-0010080-b011] Fay JC, McCullough HL, Sniegowski PD, Eisen MB (2004). Population genetic variation in gene expression is associated with phenotypic variation in *Saccharomyces cerevisiae*. Genome Biol.

[pgen-0010080-b012] Steiner HY, Naider F, Becker JM (1995). The PTR family: A new group of peptide transporters. Mol Microbiol.

[pgen-0010080-b013] Hauser M, Narita V, Donhardt AM, Naider F, Becker JM (2001). Multiplicity and regulation of genes encoding peptide transporters in *Saccharomyces cerevisiae*. Mol Membr Biol.

[pgen-0010080-b014] Stacey G, Koh S, Granger C, Becker JM (2002). Peptide transport in plants. Trends Plant Sci.

[pgen-0010080-b015] Herrera-Ruiz D, Knipp GT (2003). Current perspectives on established and putative mammalian oligopeptide transporters. J Pharm Sci.

[pgen-0010080-b016] Payne JW, Smith MW (1994). Peptide transport by micro-organisms. Adv Microb Physiol.

[pgen-0010080-b017] Manson MD, Blank V, Brade G, Higgins CF (1986). Peptide chemotaxis in *E. coli* involves the Tap signal transducer and the dipeptide permease. Nature.

[pgen-0010080-b018] Mathiopoulos C, Mueller JP, Slack FJ, Murphy CG, Patankar S (1991). A *Bacillus subtilis* dipeptide transport system expressed early during sporulation. Mol Microbiol.

[pgen-0010080-b019] Perego M, Higgins CF, Pearce SR, Gallagher MP, Hoch JA (1991). The oligopeptide transport system of *Bacillus subtilis* plays a role in the initiation of sporulation. Mol Microbiol.

[pgen-0010080-b020] Goodell EW, Higgins CF (1987). Uptake of cell wall peptides by *Salmonella typhimurium* and *Escherichia coli*. J Bacteriol.

[pgen-0010080-b021] Ringrose PS, Payne JW (1980). Peptides as antimicrobial agents. Microorganisms and nitrogen sources.

[pgen-0010080-b022] St Georgiev V (2000). Membrane transporters and antifungal drug resistance. Curr Drug Targets.

[pgen-0010080-b023] Nielsen CU, Brodin B (2003). Di/tri-peptide transporters as drug delivery targets: regulation of transport under physiological and patho-physiological conditions. Curr Drug Targets.

[pgen-0010080-b024] Becker JM, Naider F, Taylor MD, Amidon GL (1995). Fungal peptide transport as a drug delivery system. Peptide-based drug design: Controlling transport and metabolism.

[pgen-0010080-b025] Graul RC, Sadee W (1997). Sequence alignments of the H(+)–dependent oligopeptide transporter family PTR: Inferences on structure and function of the intestinal PET1 transporter. Pharm Res.

[pgen-0010080-b026] Mackenzie B, Loo DD, Fei Y, Liu WJ, Ganapathy V (1996). Mechanisms of the human intestinal H+–coupled oligopeptide transporter hPEPT1. J Biol Chem.

[pgen-0010080-b027] Chiang CS, Stacey G, Tsay YF (2004). Mechanisms and functional properties of two peptide transporters, AtPTR2 and fPTR2. J Biol Chem.

[pgen-0010080-b028] Alagramam K, Naider F, Becker JM (1995). A recognition component of the ubiquitin system is required for peptide transport in *Saccharomyces cerevisiae*. Mol Microbiol.

[pgen-0010080-b029] Island MD, Naider F, Becker JM (1987). Regulation of dipeptide transport in *Saccharomyces cerevisiae* by micromolar amino acid concentrations. J Bacteriol.

[pgen-0010080-b030] Varshavsky A (1997). The N-end rule pathway of protein degradation. Genes Cells.

[pgen-0010080-b031] Varshavsky A (1996). The N-end rule: Functions, mysteries, uses. Proc Natl Acad Sci U S A.

[pgen-0010080-b032] Du F, Navarro-Garcia F, Xia Z, Tasaki T, Varshavsky A (2002). Pairs of dipeptides synergistically activate the binding of substrate by ubiquitin ligase through dissociation of its autoinhibitory domain. Proc Natl Acad Sci U S A.

[pgen-0010080-b033] Turner GC, Du F, Varshavsky A (2000). Peptides accelerate their uptake by activating a ubiquitin-dependent proteolytic pathway. Nature.

[pgen-0010080-b034] Byrd C, Turner GC, Varshavsky A (1998). The N-end rule pathway controls the import of peptides through degradation of a transcriptional repressor. EMBO J.

[pgen-0010080-b035] Bochner BR, Gadzinski P, Panomitros E (2001). Phenotype microarrays for high-throughput phenotypic testing and assay of gene function. Genome Res.

[pgen-0010080-b036] Bochner BR (2003). New technologies to assess genotype-phenotype relationships. Nat Rev Genet.

[pgen-0010080-b037] Tanzer MM, Arst HN, Skalchunes AR, Coffin M, Darveaux BA (2003). Global nutritional profiling for mutant and chemical mode-of-action analysis in filamentous fungi. Funct Integr Genomics.

[pgen-0010080-b038] Greig D, Travisano M, Louis EJ, Borts RH (2003). A role for the mismatch repair system during incipient speciation in *Saccharomyces*. J Evol Biol.

[pgen-0010080-b039] Bochner BR (1989). 'Breathprints' at the microbial level. ASM News.

[pgen-0010080-b040] LaRue TA, Spencer JF (1967). The utilization of D-amino acids by yeasts. Can J Microbiol.

[pgen-0010080-b041] De Deken RH (1962). Pathway of arginine biosynthesis in yeast. Biochem Biophys Res Commun.

[pgen-0010080-b042] Cooper TG, Brambl R, Marzluf GA (1996). Regulation of allantoin catabolism in *Saccharomyces cerevisiae*. The mycota III: Biochemistry and molecular biology.

[pgen-0010080-b043] Chisholm VT, Lea HZ, Rai R, Cooper TG (1987). Regulation of allantoate transport in wild-type and mutant strains of *Saccharomyces cerevisiae*. J Bacteriol.

[pgen-0010080-b044] Greth ML, Chevallier MR, Lacroute F (1977). Ureidosuccinic acid permeation in *Saccharomyces cerevisiae*. Biochim Biophys Acta.

[pgen-0010080-b045] Bader GD, Heilbut A, Andrews B, Tyers M, Hughes T (2003). Functional genomics and proteomics: charting a multidimensional map of the yeast cell. Trends Cell Biol.

[pgen-0010080-b046] Tong AH, Lesage G, Bader GD, Ding H, Xu H (2004). Global mapping of the yeast genetic interaction network. Science.

[pgen-0010080-b047] Gavin AC, Bosche M, Krause R, Grandi P, Marzioch M (2002). Functional organization of the yeast proteome by systematic analysis of protein complexes. Nature.

[pgen-0010080-b048] Ho Y, Gruhler A, Heilbut A, Bader GD, Moore L (2002). Systematic identification of protein complexes in Saccharomyces cerevisiae by mass spectrometry. Nature.

[pgen-0010080-b049] Lee TI, Rinaldi NJ, Robert F, Odom DT, Bar-Joseph Z (2002). Transcriptional regulatory networks in *Saccharomyces cerevisiae*. Science.

[pgen-0010080-b050] Herrmann V, Knack I, Rohm KH (1978). Yeast dipeptidase: Active site mapping by kinetic studies with substrates and substrate analogs. Hoppe Seylers Z Physiol Chem.

[pgen-0010080-b051] Turoscy V, Cooper TG (1987). Ureidosuccinate is transported by the allantoate transport system in *Saccharomyces cerevisiae*. J Bacteriol.

[pgen-0010080-b052] Rai R, Daugherty JR, Tate JJ, Buford TD, Cooper TG (2004). Synergistic operation of four cis-acting elements mediate high level DAL5 transcription in *Saccharomyces cerevisiae*. FEMS Yeast Res.

[pgen-0010080-b053] Rai R, Genbauffe F, Lea HZ, Cooper TG (1987). Transcriptional regulation of the DAL5 gene in *Saccharomyces cerevisiae*. J Bacteriol.

[pgen-0010080-b054] Bon EP, Carvajal E, Stanbrough M, Rowen D, Magasanik B (1997). Asparaginase II of *Saccharomyces cerevisiae.* GLN3/URE2 regulation of a periplasmic enzyme. Appl Biochem Biotechnol.

[pgen-0010080-b055] Dunlop PC, Meyer GM, Ban D, Roon RJ (1978). Characterization of two forms of asparaginase in *Saccharomyces cerevisiae*. J Biol Chem.

[pgen-0010080-b056] Dunlop PC, Roon RJ, Even HL (1976). Utilization of D-asparagine by *Saccharomyces cerevisiae*. J Bacteriol.

[pgen-0010080-b057] Lashkari DA, DeRisi JL, McCusker JH, Namath AF, Gentile C (1997). Yeast microarrays for genome wide parallel genetic and gene expression analysis. Proc Natl Acad Sci U S A.

[pgen-0010080-b058] Howard JB, Carpenter FH (1972). L-asparaginase from *Erwinia carotovora.* Substrate specificity and enzymatic properties. J Biol Chem.

[pgen-0010080-b059] Ausubel FM, Brent R, Kingston RE, Moore DD, Seidman JG (1988). Current protocols in molecular biology.

[pgen-0010080-b060] Hauser M, Kauffman S, Naider F, Becker JM (2005). Substrate preference is altered by mutations in the fifth transmembrane domain of Ptr2p, the di/tri-peptide transporter of *Saccharomyces cerevisiae*. Mol Membr Biol.

[pgen-0010080-b061] Stade K, Ford CS, Guthrie C, Weis K (1997). Exportin 1 (Crm1p) is an essential nuclear export factor. Cell.

[pgen-0010080-b062] Wach A, Brachat A, Alberti-Segui C, Rebischung C, Philippsen P (1997). Heterologous HIS3 marker and GFP reporter modules for PCR-targeting in *Saccharomyces cerevisiae*. Yeast.

[pgen-0010080-b063] Christianson TW, Sikorski RS, Dante M, Shero JH, Hieter P (1992). Multifunctional yeast high-copy-number shuttle vectors. Gene.

[pgen-0010080-b064] Guldener U, Heck S, Fielder T, Beinhauer J, Hegemann JH (1996). A new efficient gene disruption cassette for repeated use in budding yeast. Nucleic Acids Res.

[pgen-0010080-b065] Goldstein AL, McCusker JH (1999). Three new dominant drug resistance cassettes for gene disruption in *Saccharomyces cerevisiae*. Yeast.

[pgen-0010080-b066] Ito H, Fukuda Y, Murata K, Kimura A (1983). Transformation of intact yeast cells treated with alkali cations. J Bacteriol.

[pgen-0010080-b067] Saldanha AJ (2004). Java Treeview—Extensible visualization of microarray data. Bioinformatics.

[pgen-0010080-b068] Connelly C, Hieter P (1996). Budding yeast SKP1 encodes an evolutionarily conserved kinetochore protein required for cell cycle progression. Cell.

[pgen-0010080-b069] De Boer M, Bebelman JP, Goncalves PM, Maat J, Van Heerikhuizen H (1998). Regulation of expression of the amino acid transporter gene BAP3 in *Saccharomyces cerevisiae*. Mol Microbiol.

[pgen-0010080-b070] Rose MD, Winston F, Hieter P (1990). Methods in yeast genetics: A laboratory course manual.

