# Supplementary figures and images for: Harnessing Natural Diversity to Probe Metabolic Pathways
Source: PLoS Genet. 2005 Dec 30;1(6):e80. doi: 10.1371/journal.pgen.0010080 (PMC1342634; doi:10.1371/journal.pgen.0010080)

**YAT7**


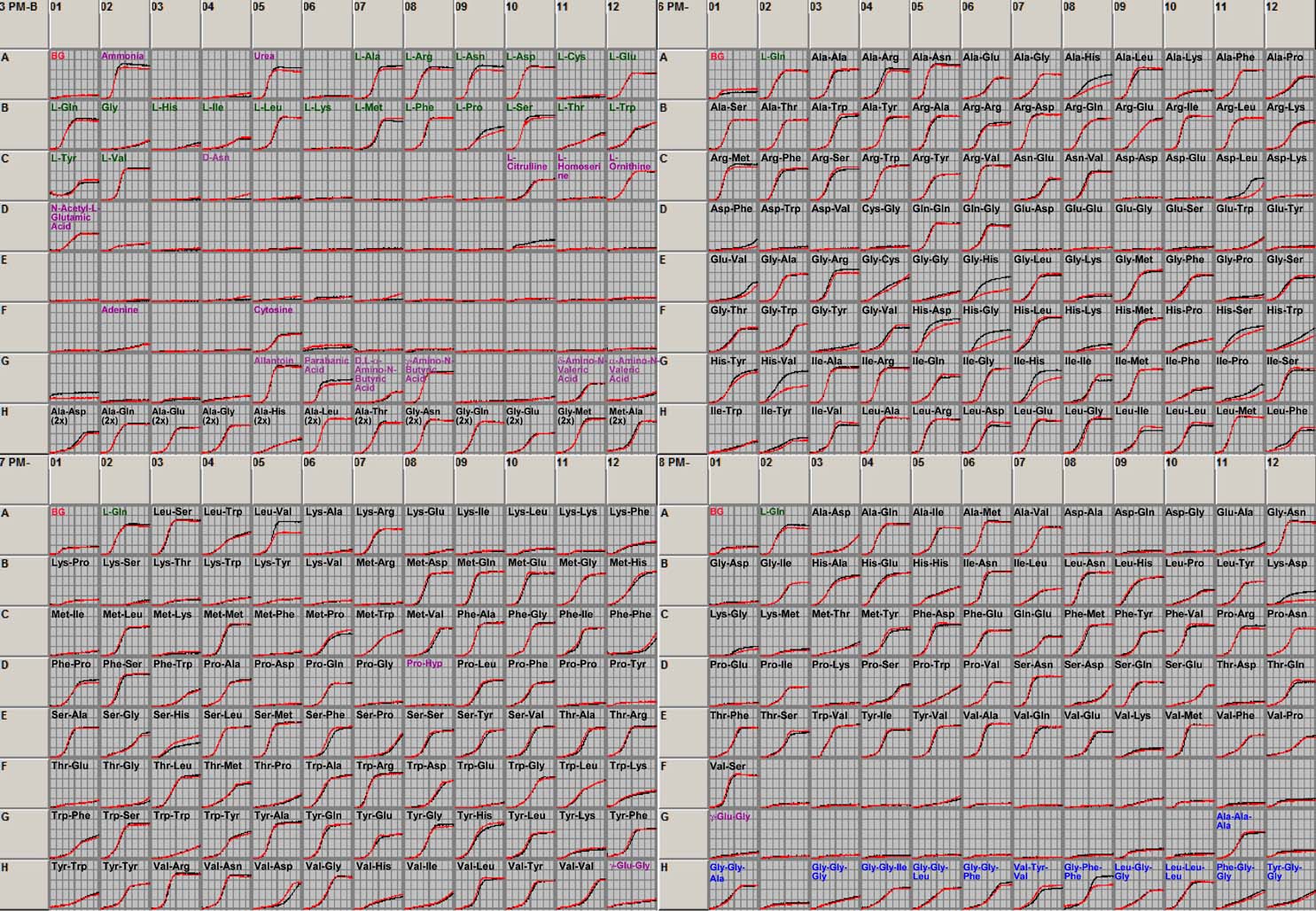


**RM3**


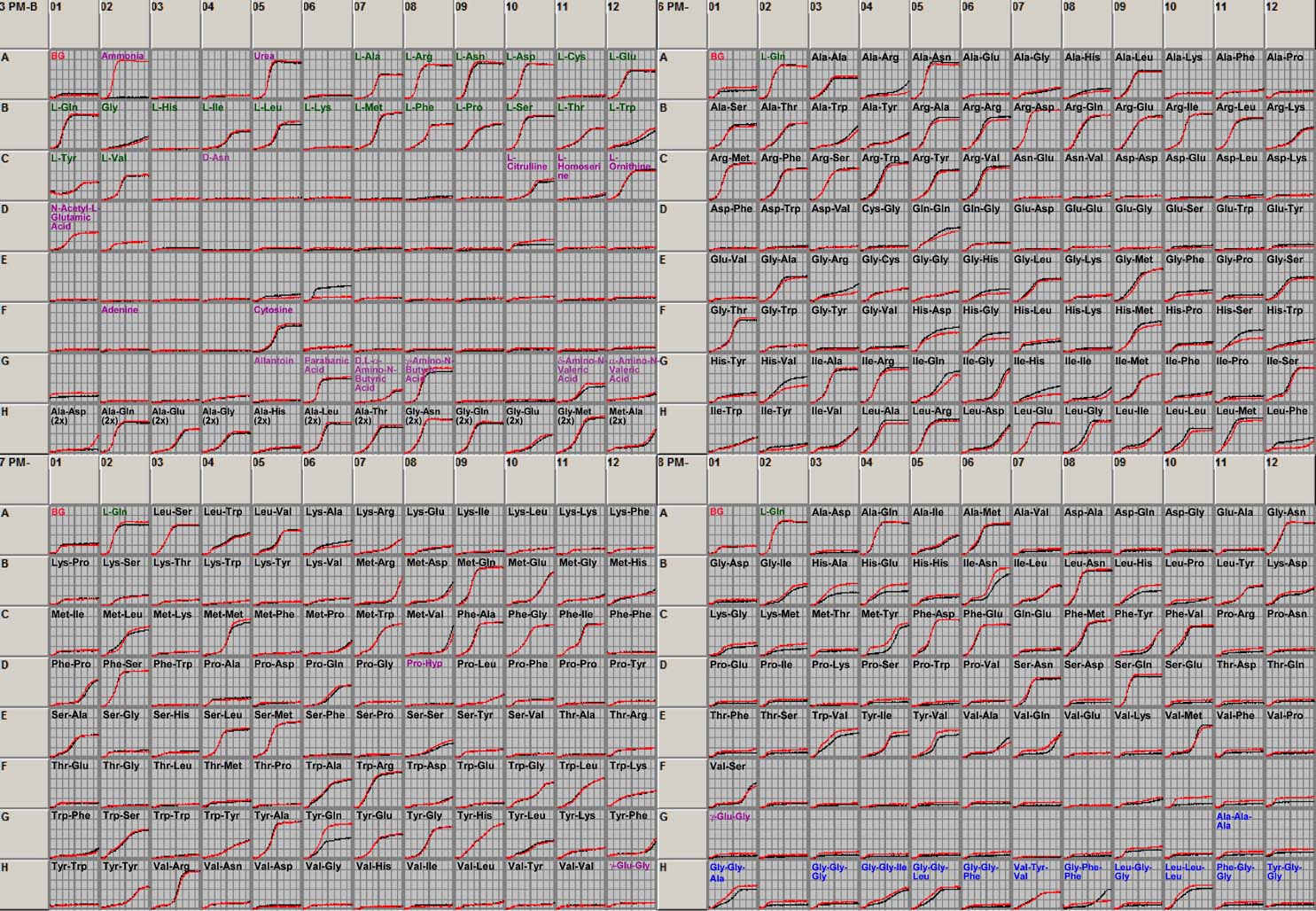


**RM8**


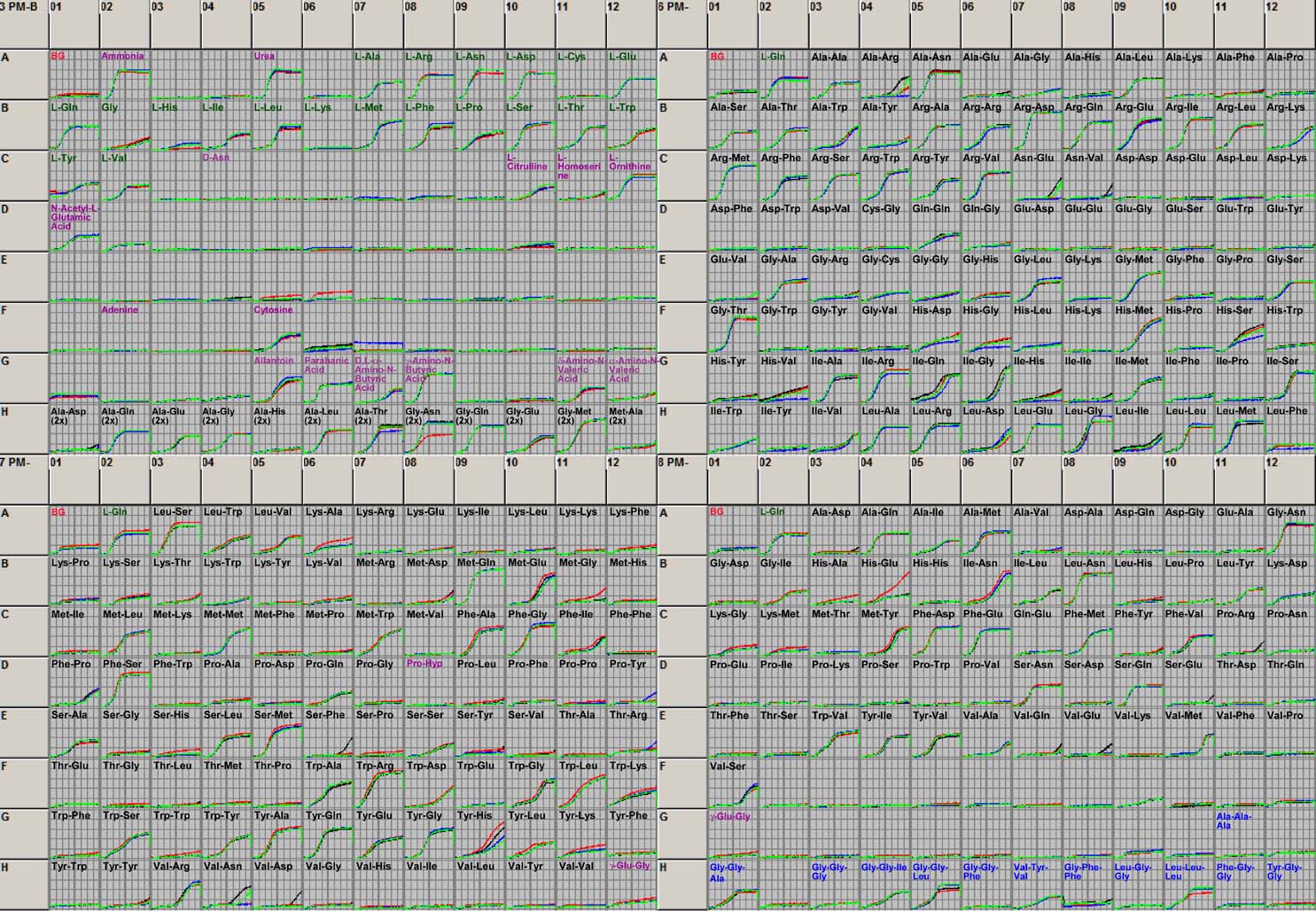


**Y55**


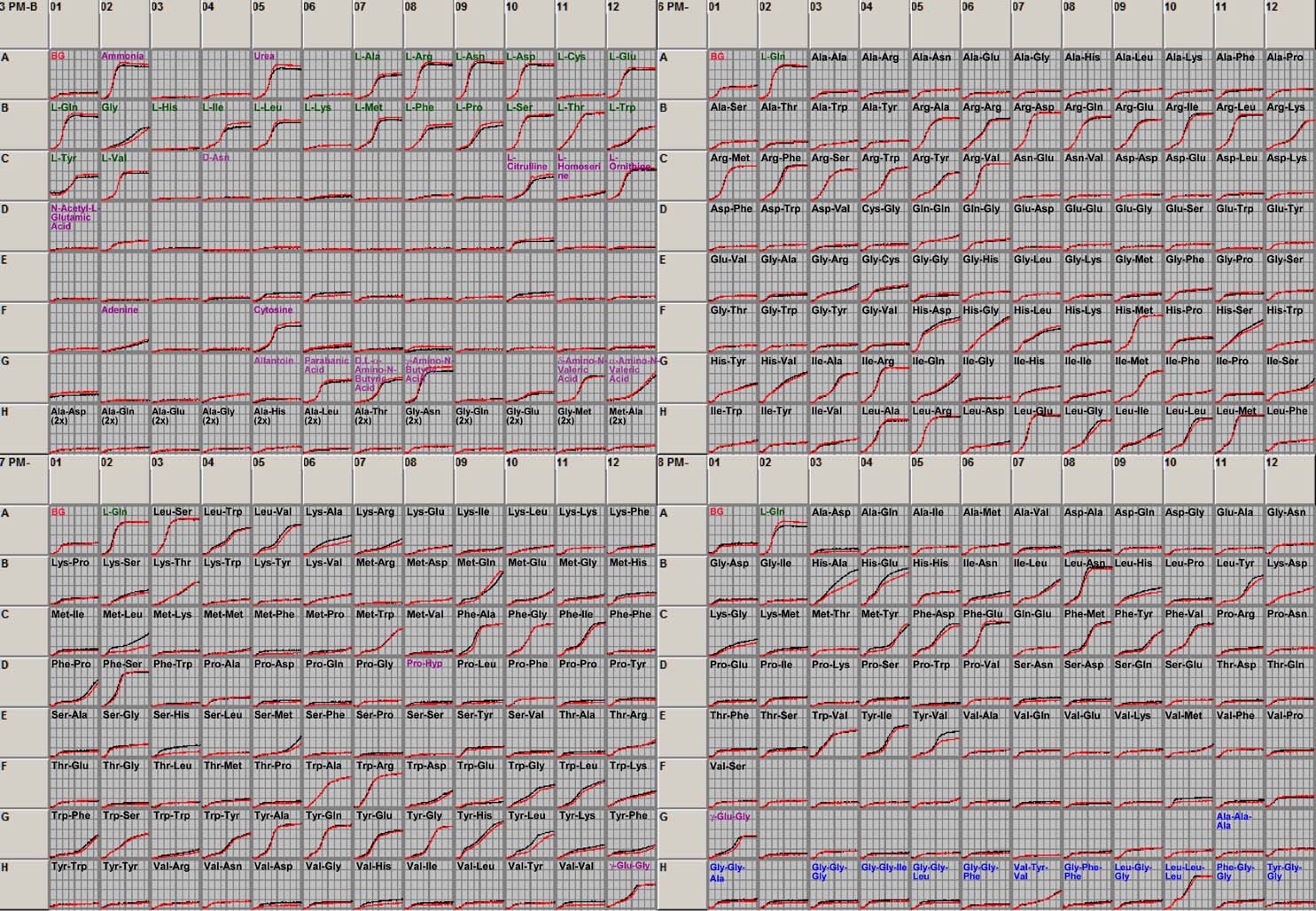


**S288c**


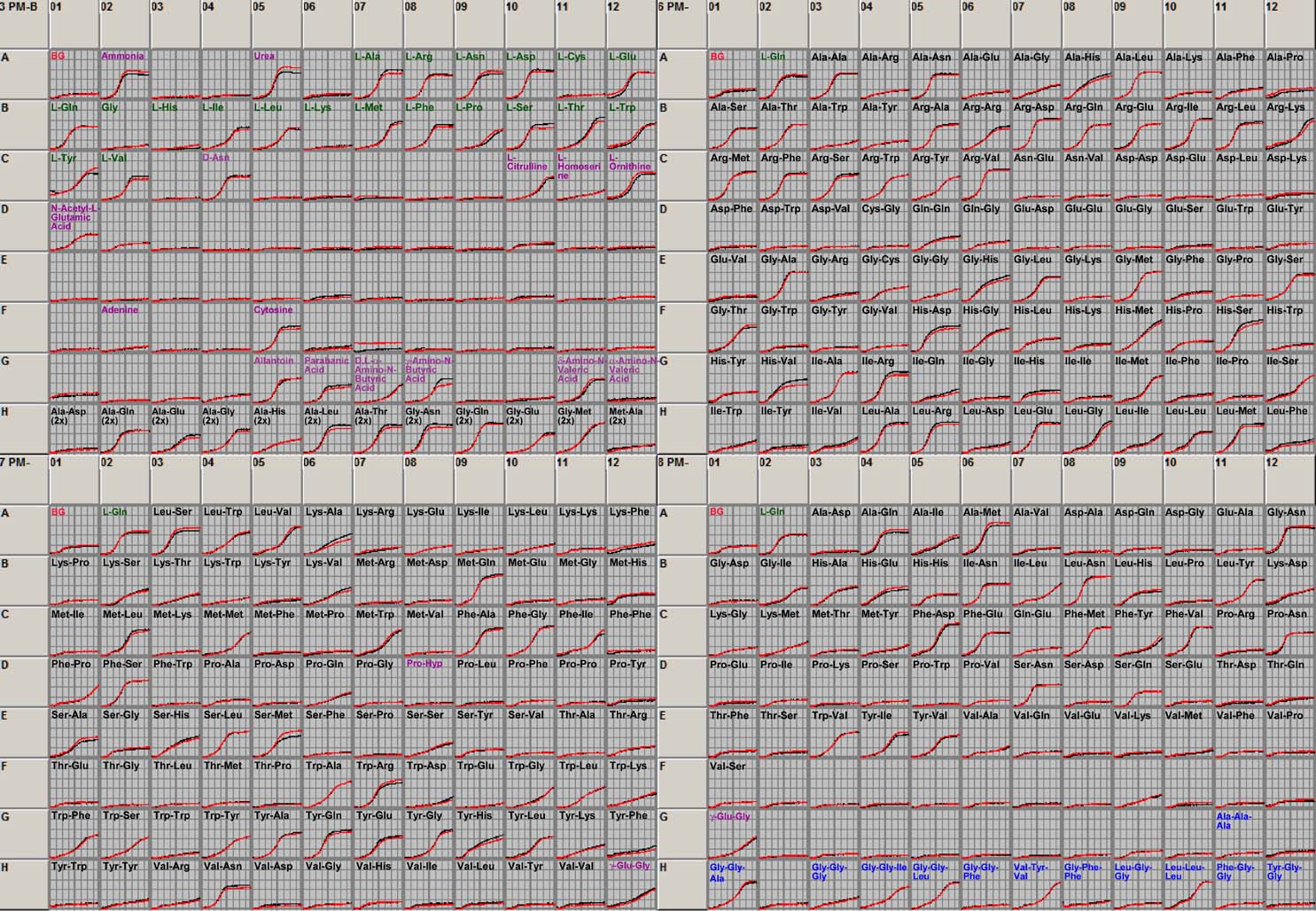


**W303**


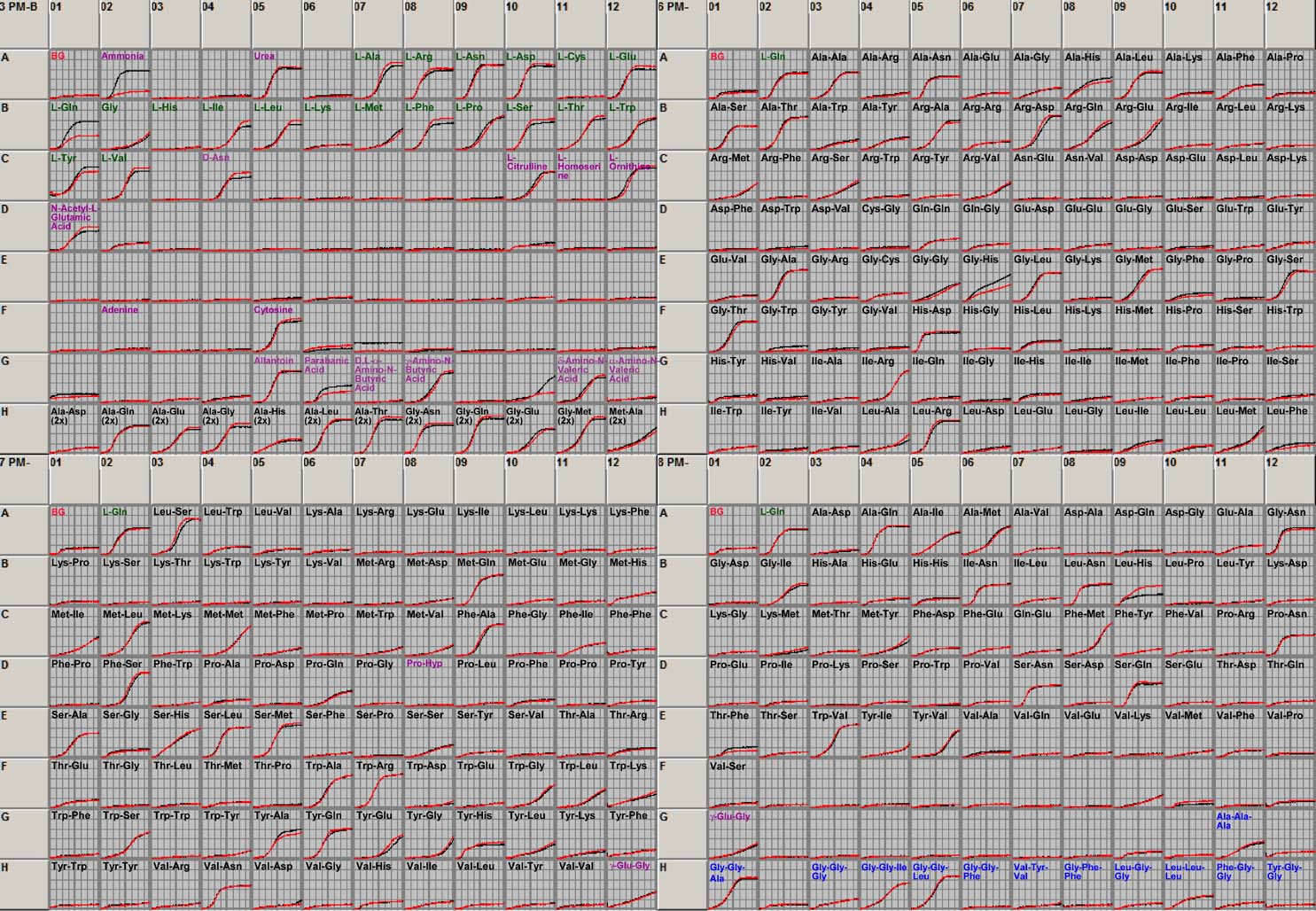

Supplement: Dataset S1 — Each image provides the signal curves generated by the OmniLog software in each well of the four 96-well plates that constitute the PM nitrogen source utilization assay (see Dataset S4 for full listing of plate contents). The x-axis of each signal curve represents the 48-h time course. The y-axis represents the signal intensity, an indirect measure of growth. The different colored lines used for the signal curves represent independent replicates of the PM assay. (2.1 MB DOC) [file pgen.0010080.sd001.doc]

**YAT7-*cup9*Δ**


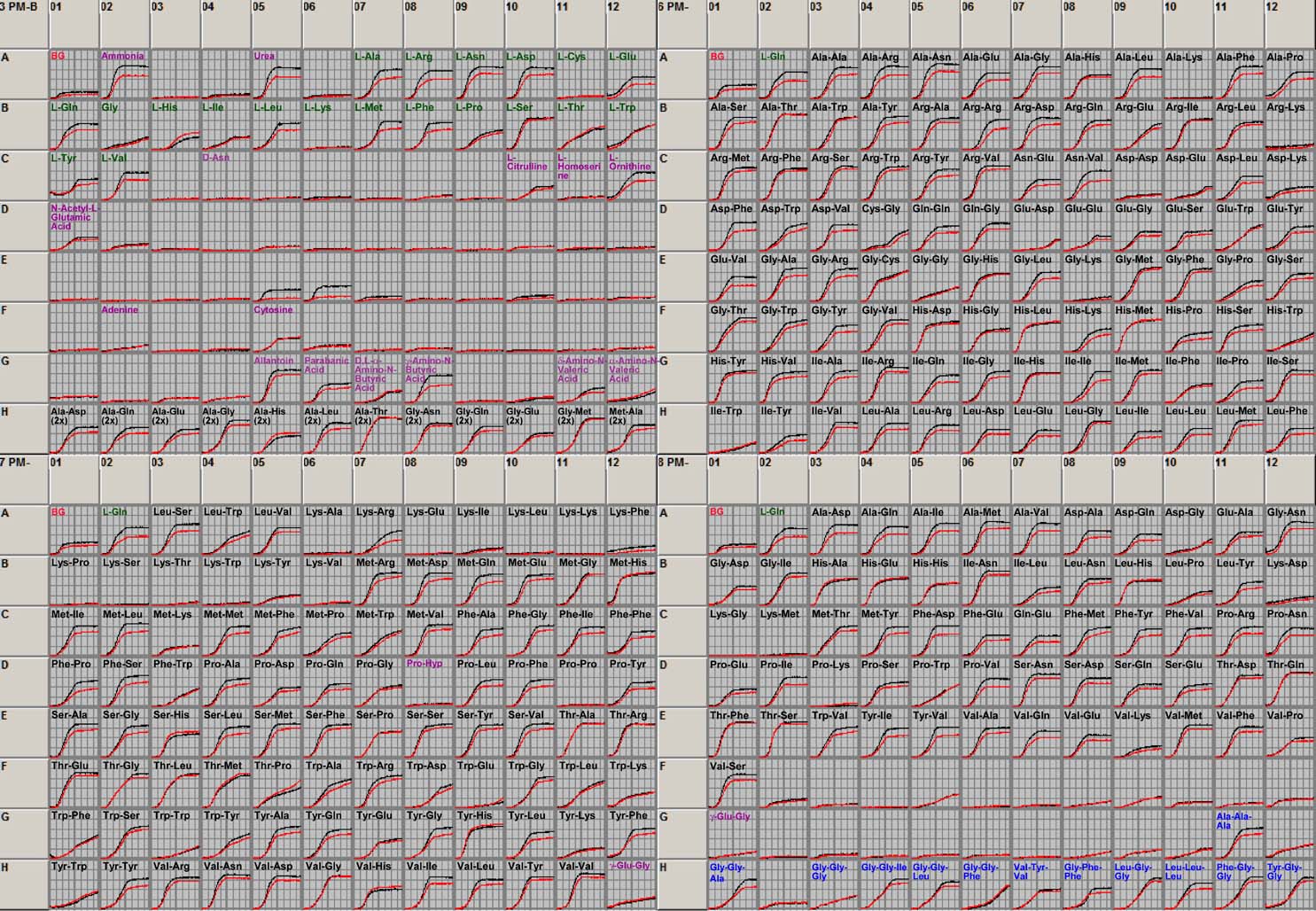


**RM8-*cup9*Δ**


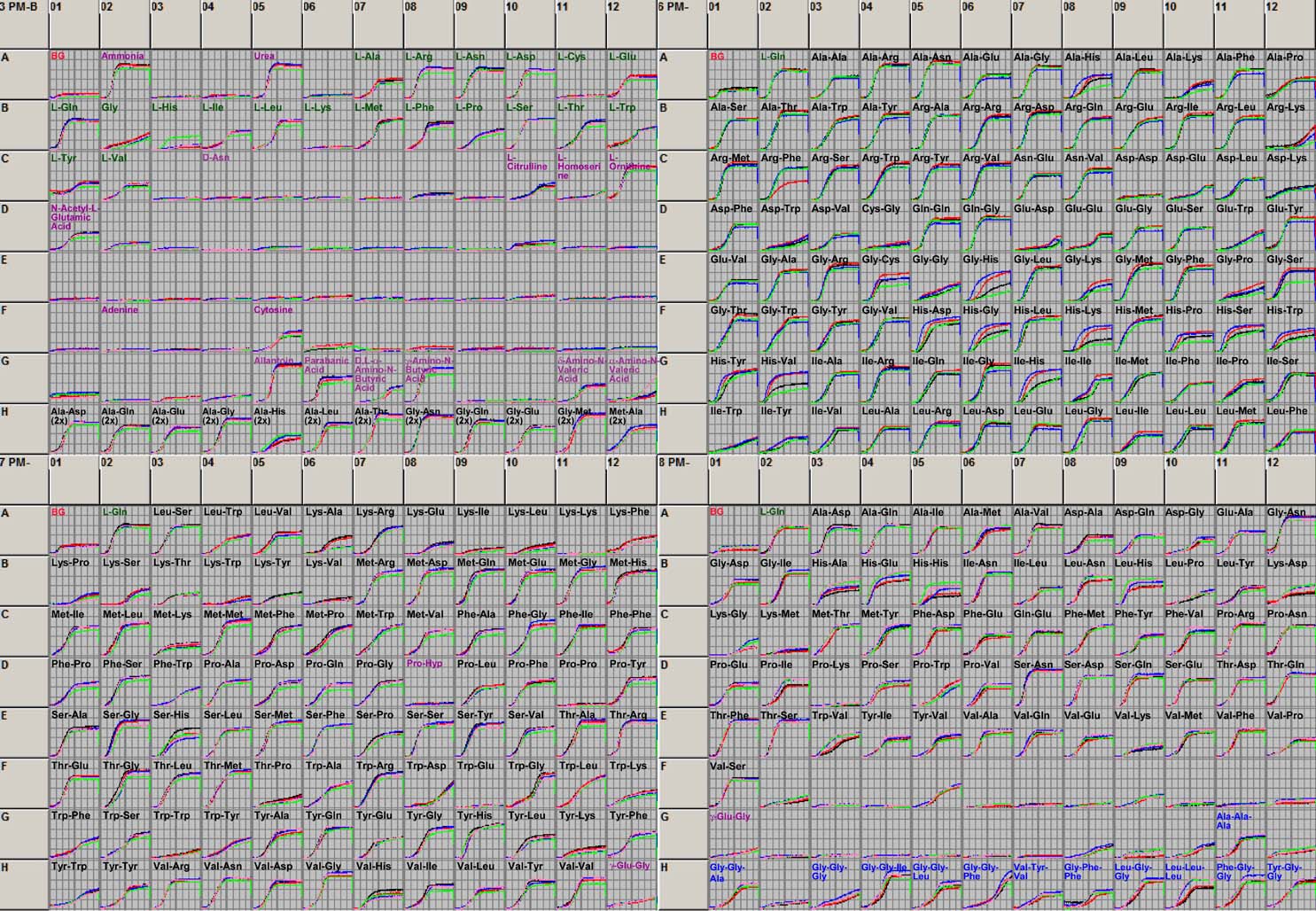


**W303-*cup9*Δ**

***
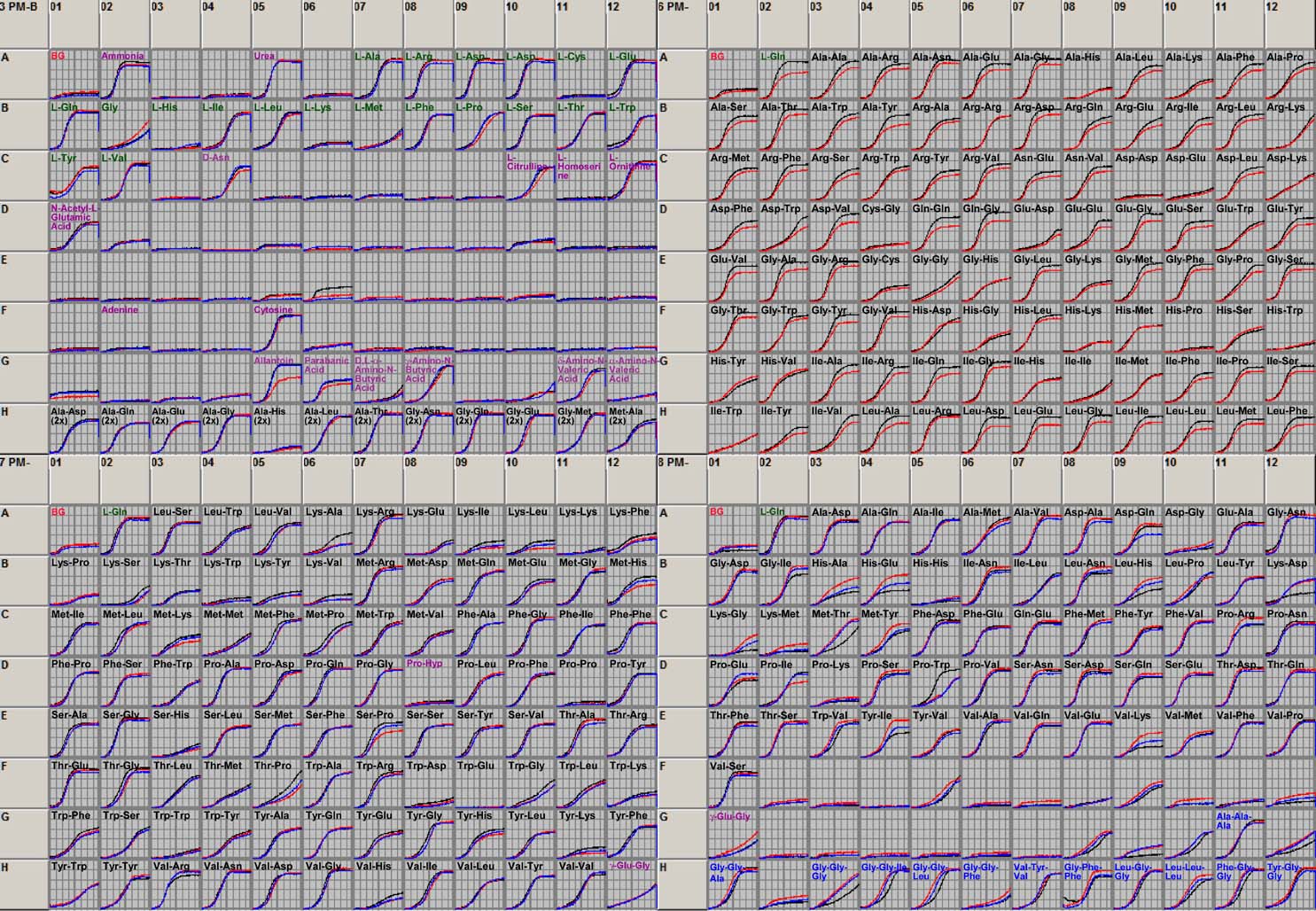
***

**YAT7-*ptr2*Δ**


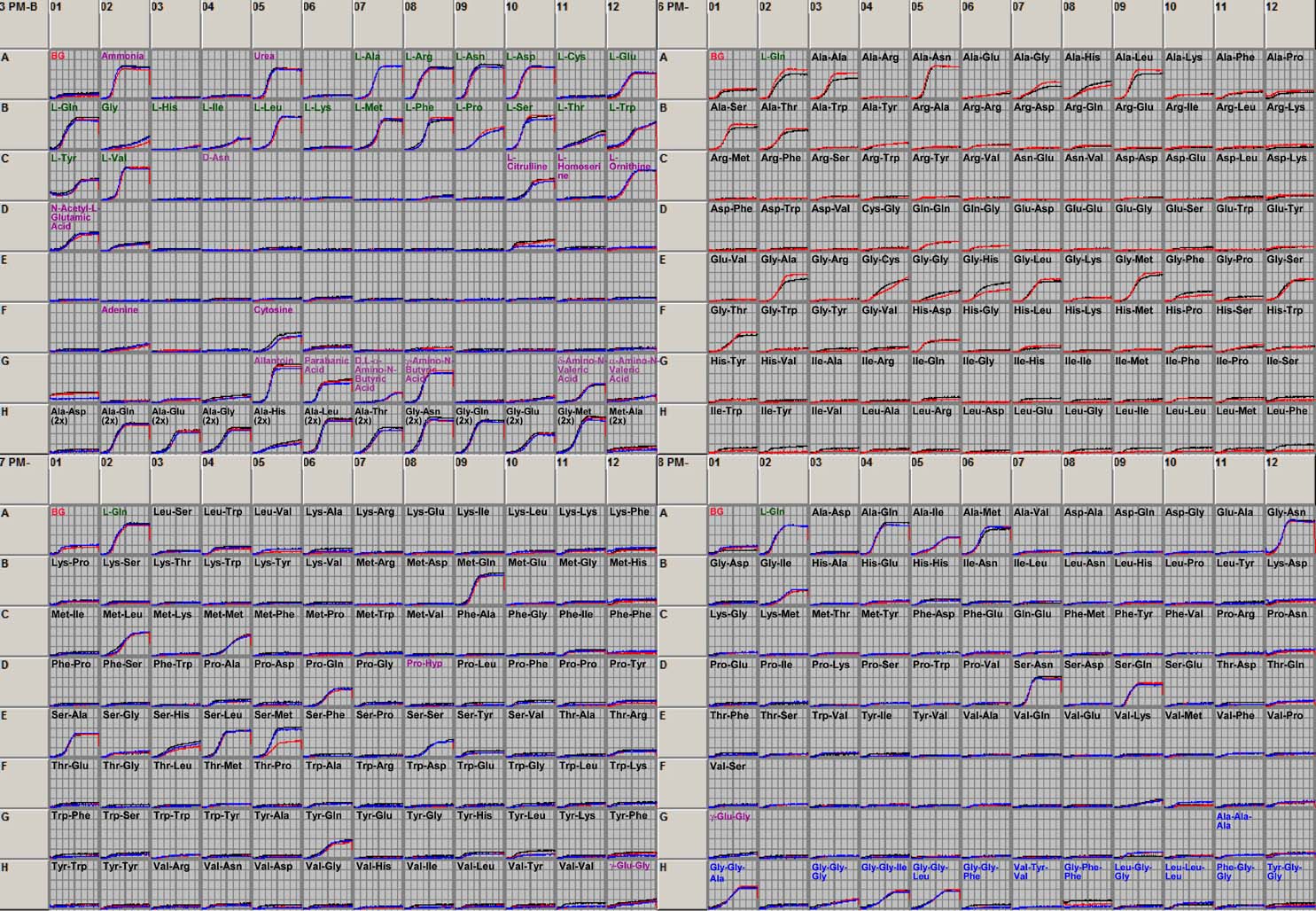


**RM8-*ptr2*Δ**


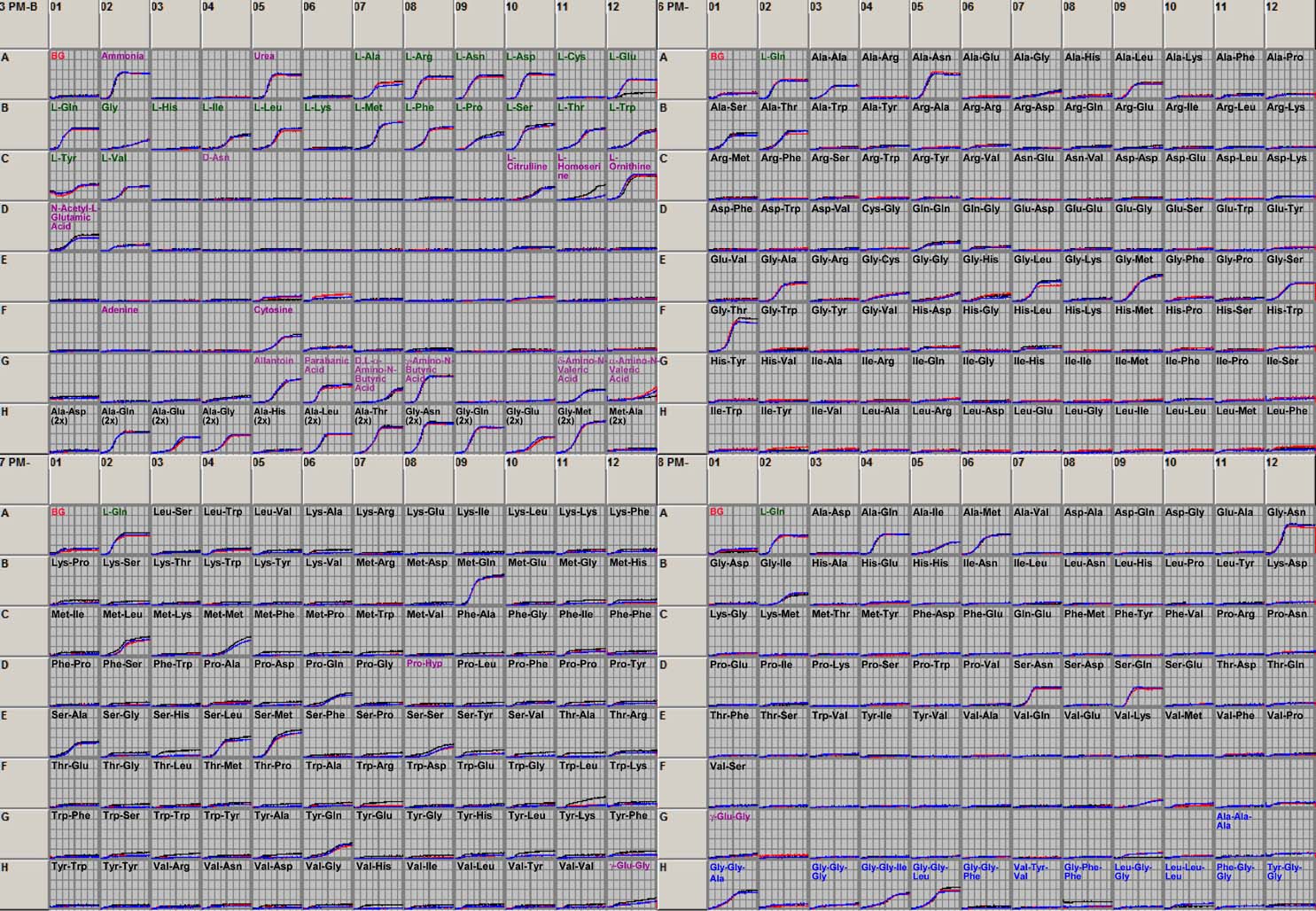


**W303-*ptr2*Δ**


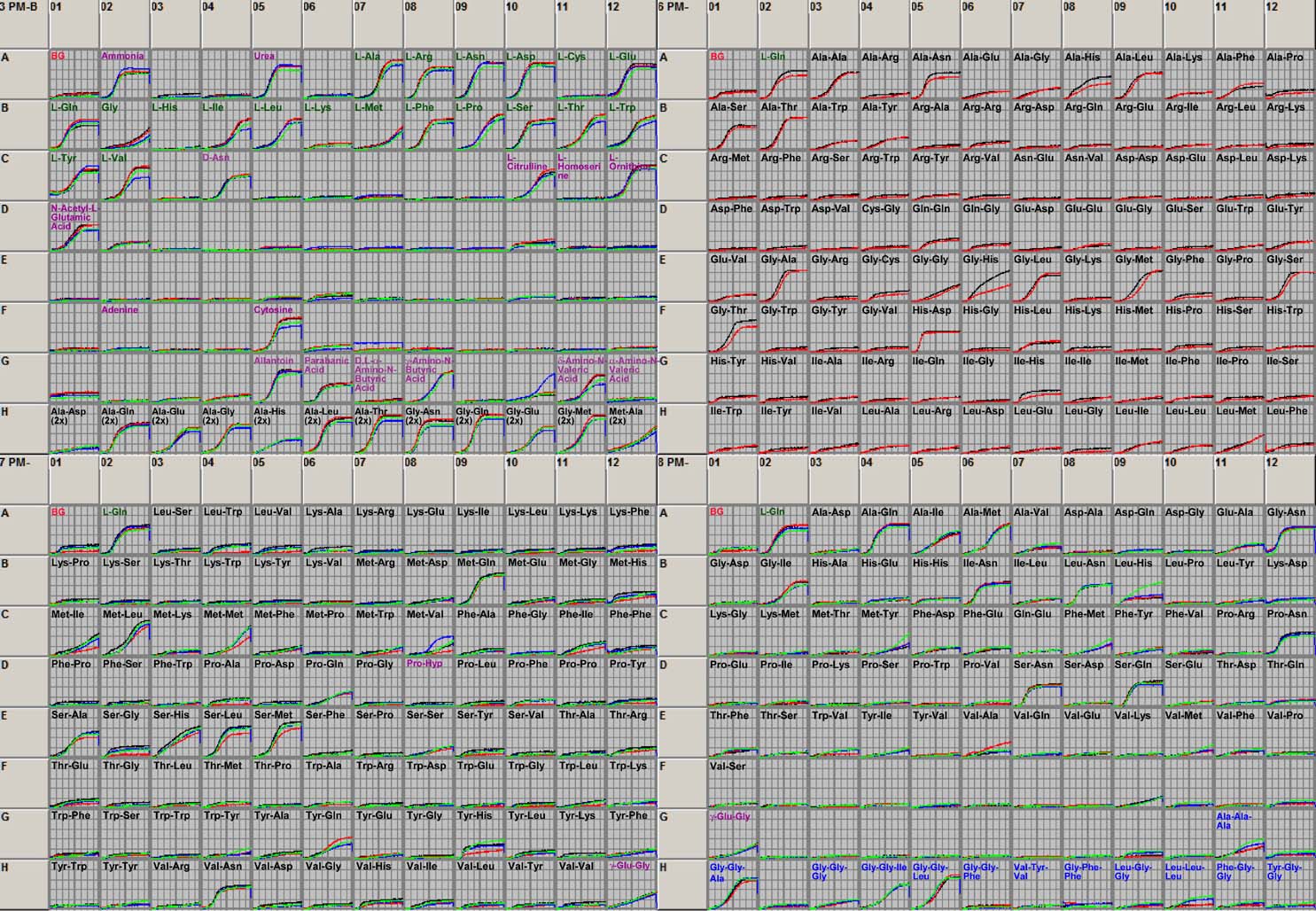

Supplement: Dataset S2 — See Dataset S1 legend for details. (2.2 MB DOC) [file pgen.0010080.sd002.doc]

**RM8**

**
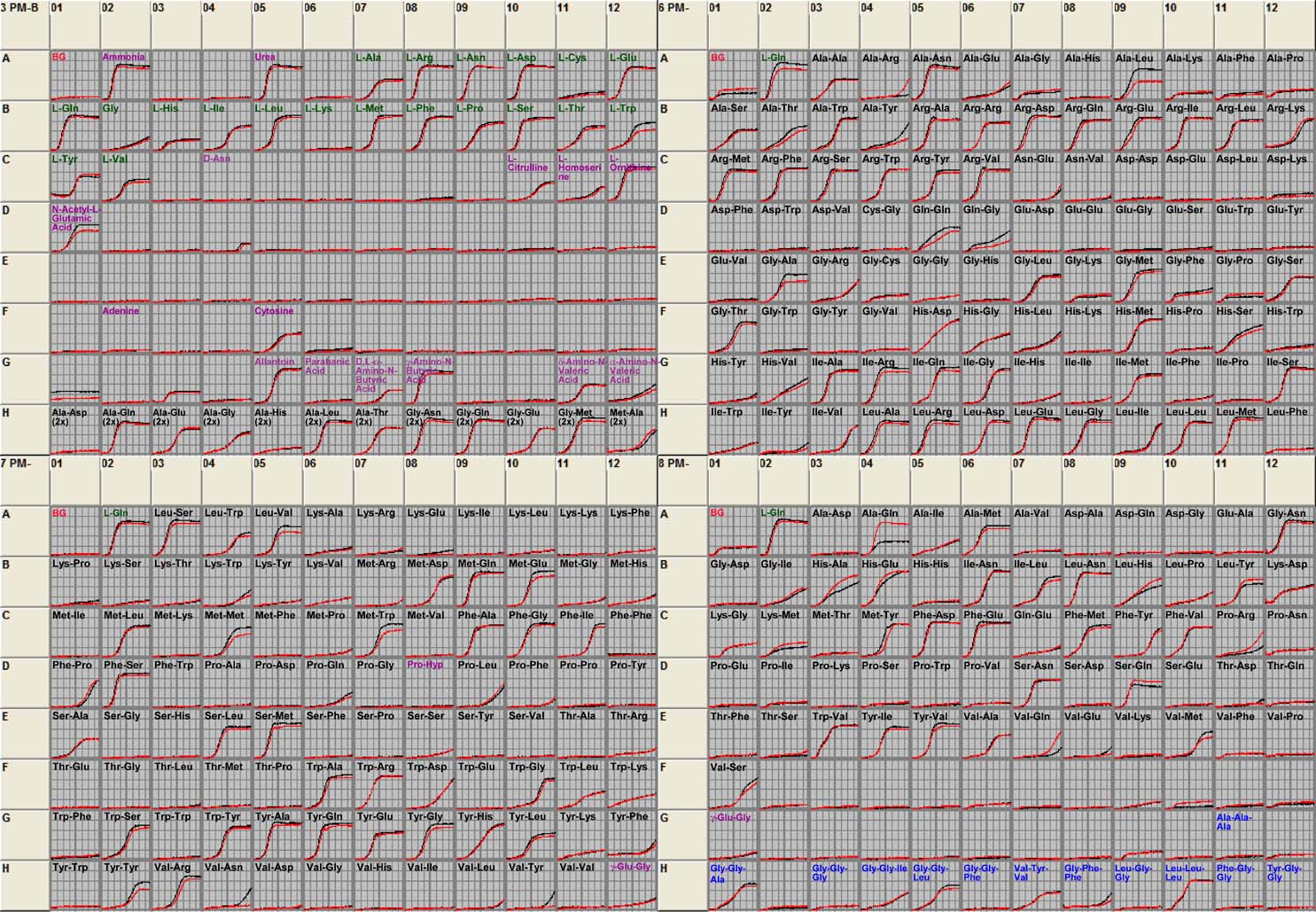
**

**RM8-*dal5*Δ**

**
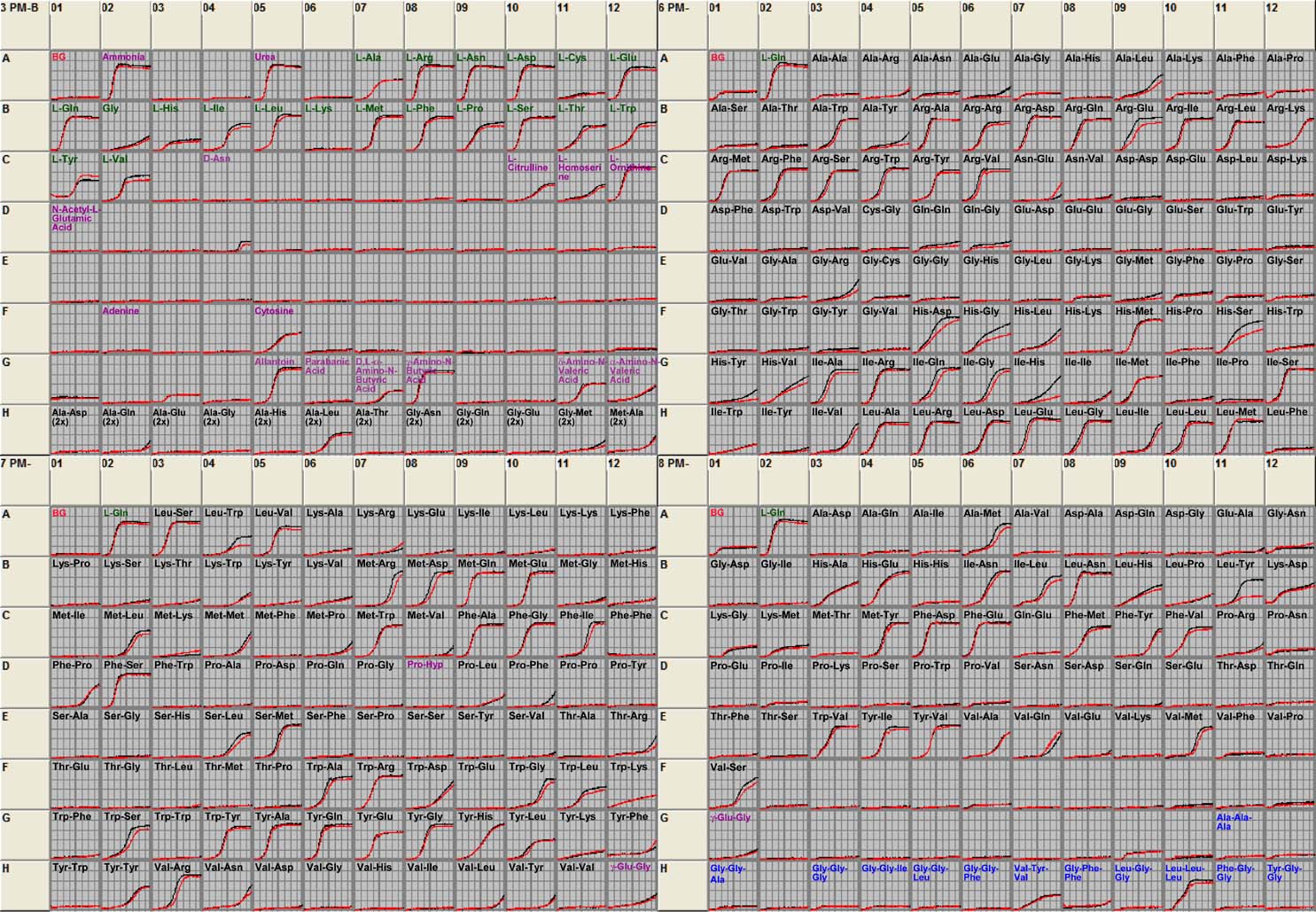
**

**RM8-*ptr2*Δ*dal5*Δ**

**
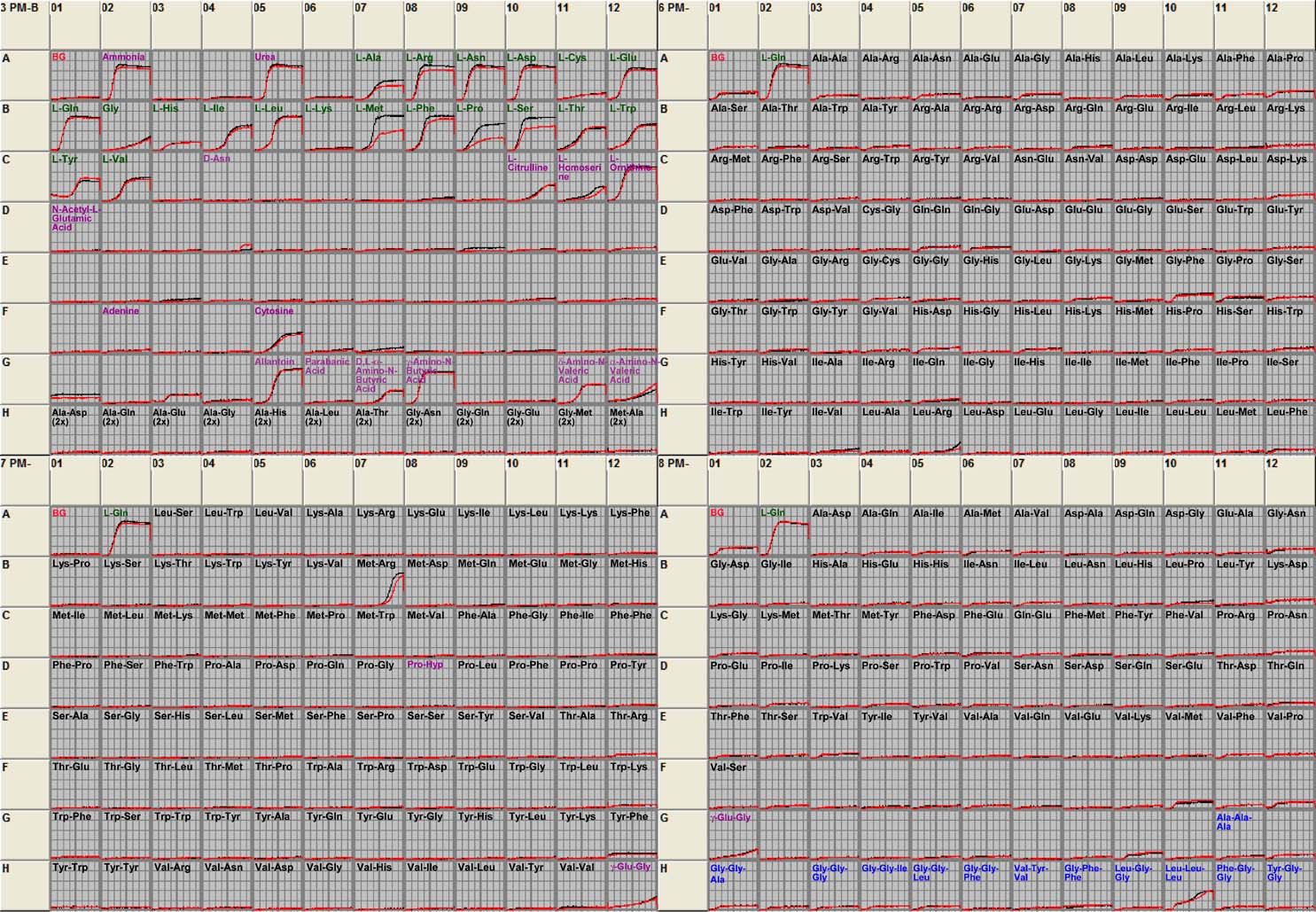
**

**Y55**

**
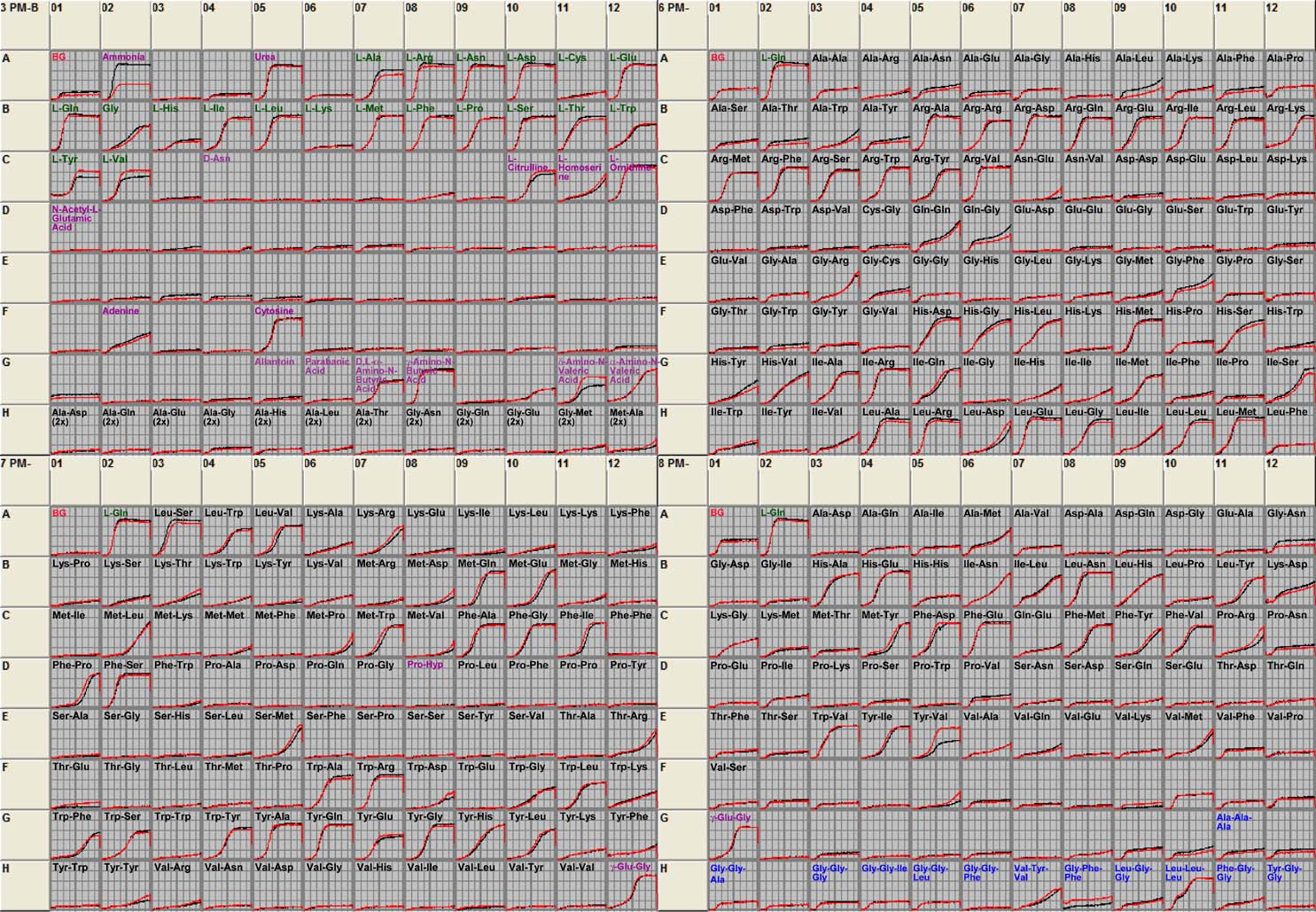
**

**Y55-*dal5*Δ**

**
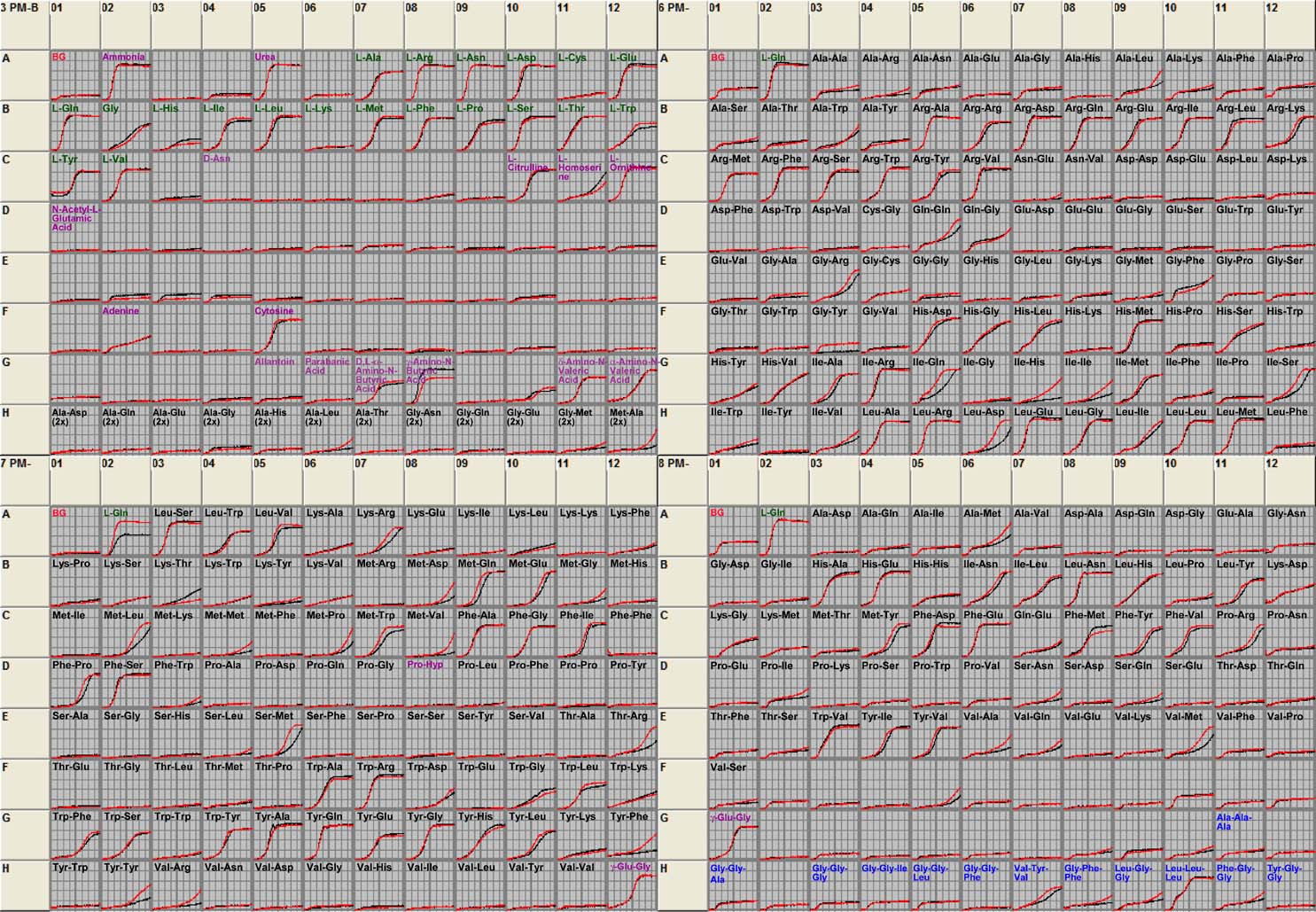
**

**Y55-*ptr2*Δ**

**
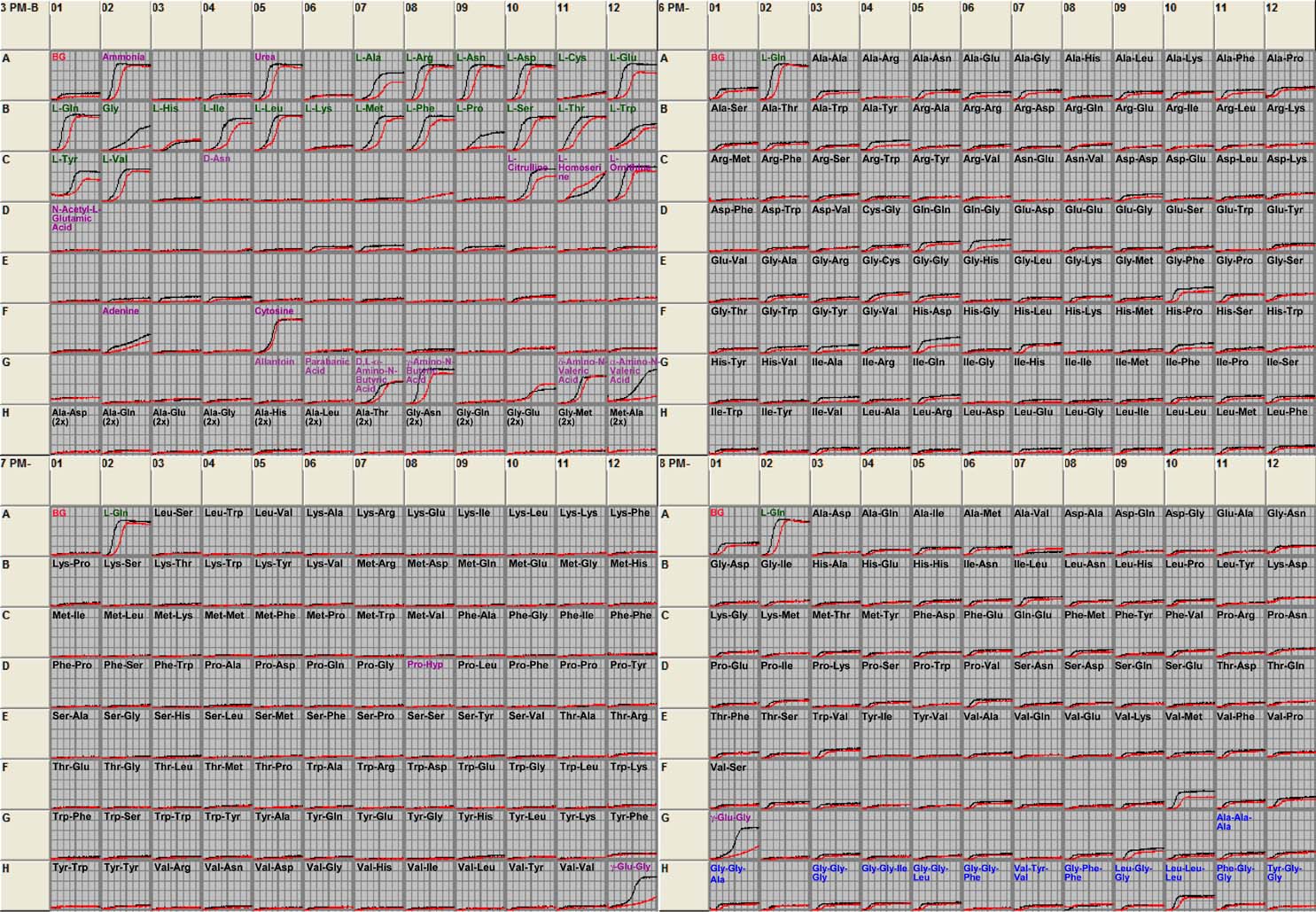
**

**W303-*ptr2*Δ*dal5*Δ**

**
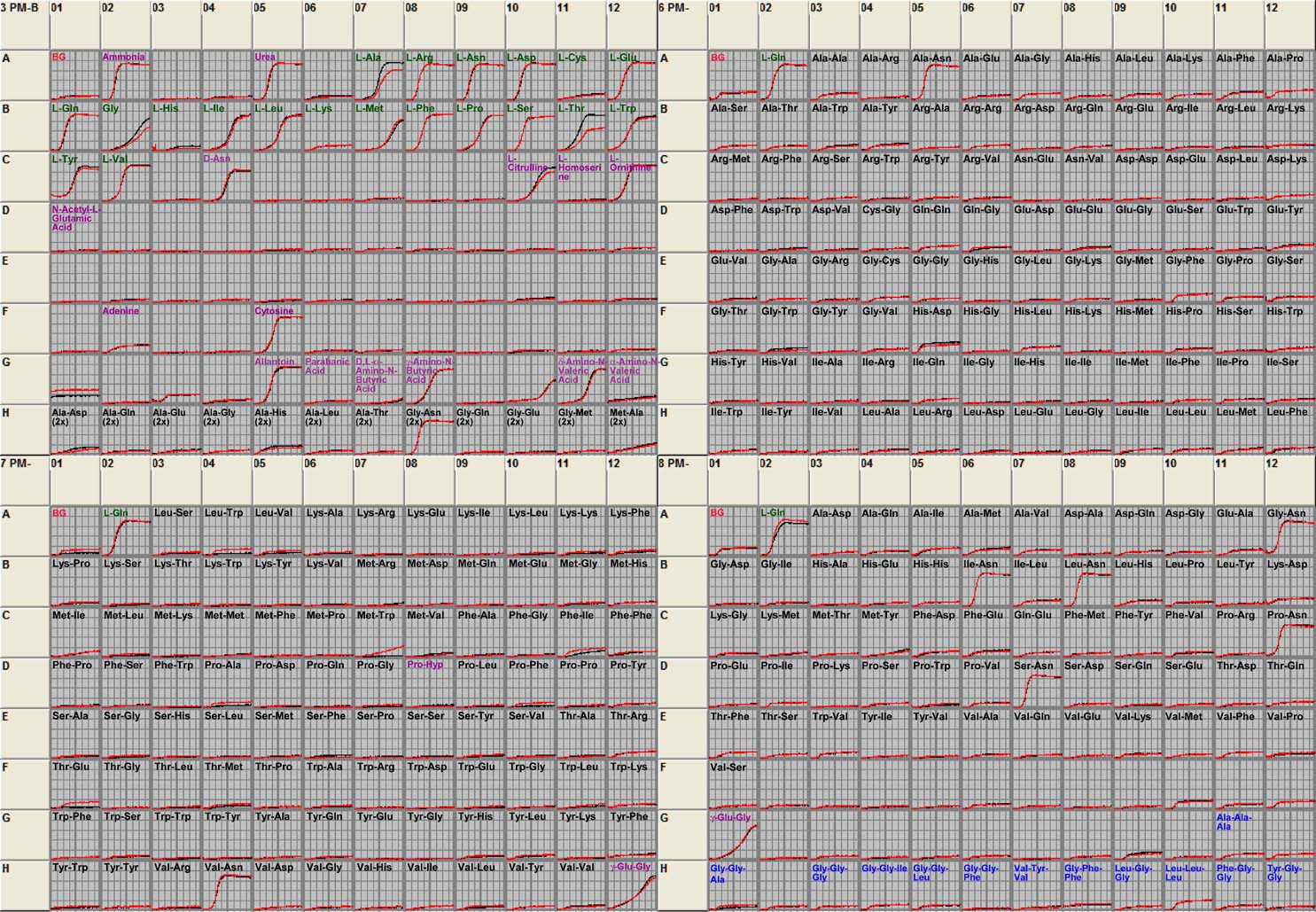
**

**W303-*ptr2*Δ (vector)**

**
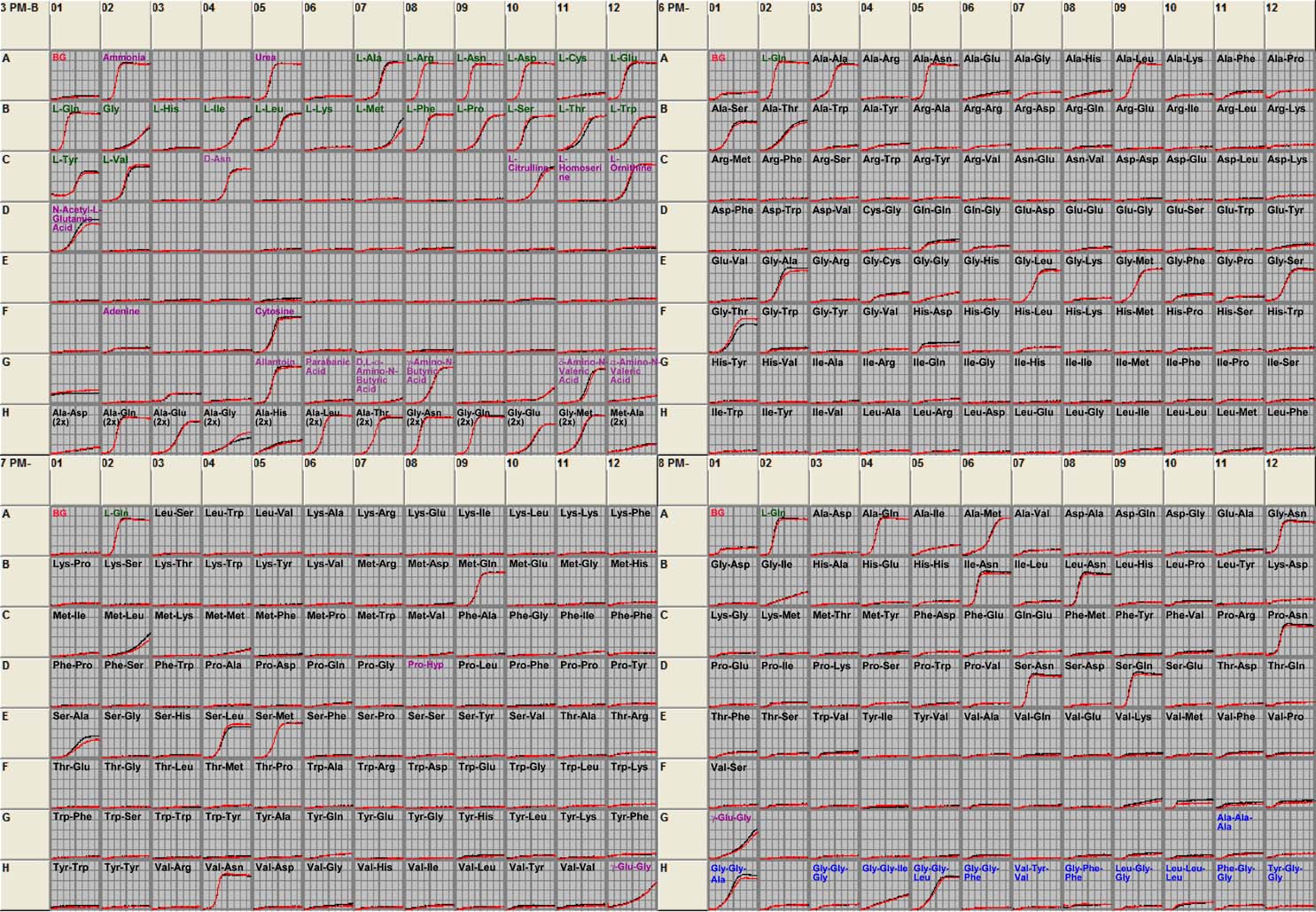
**

**W303-*ptr2*Δ (*DAL5*)**

**
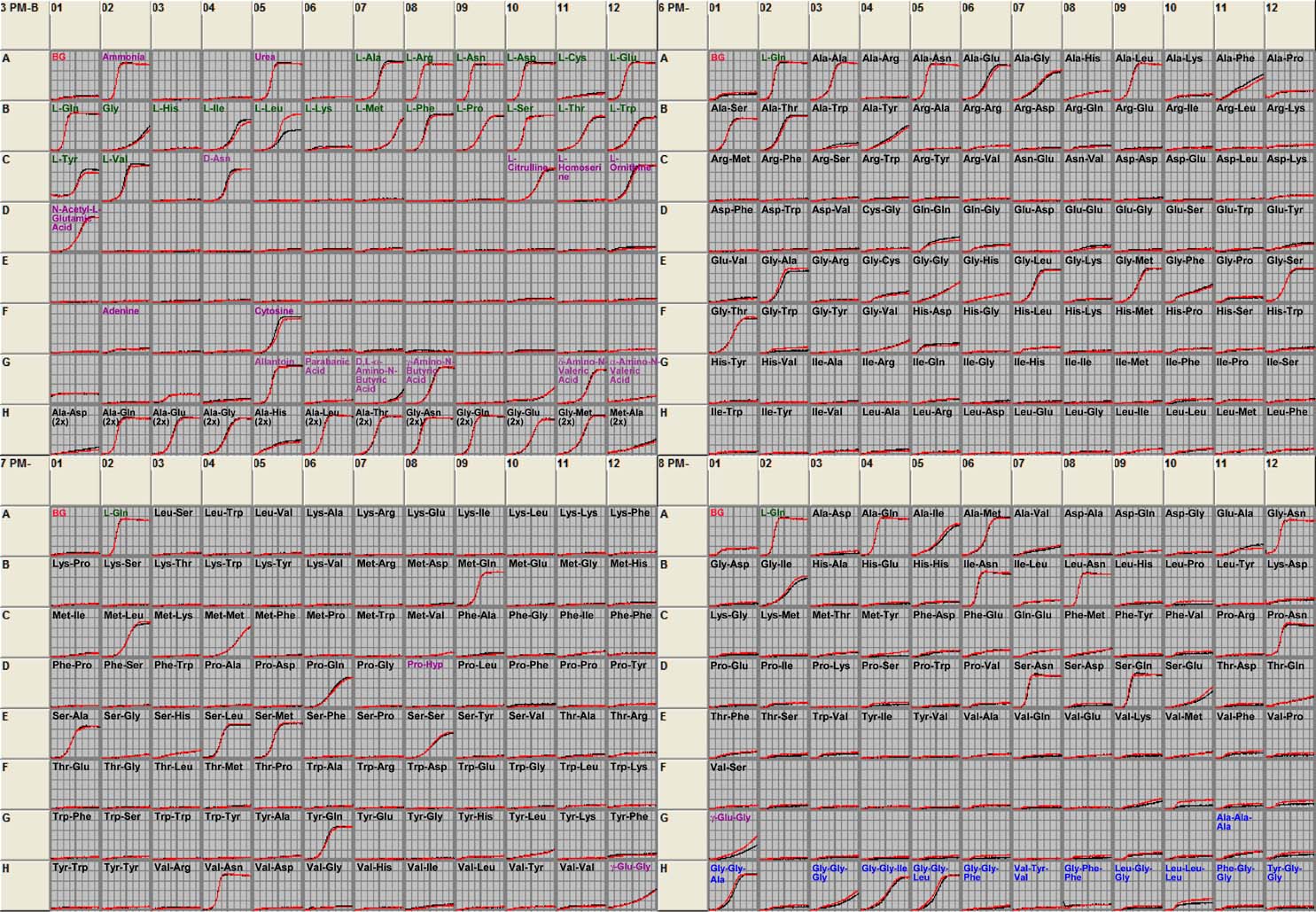
**

Supplement: Dataset S3 — See Dataset S1 legend for details. (3.1 MB DOC) [file pgen.0010080.sd003.doc]

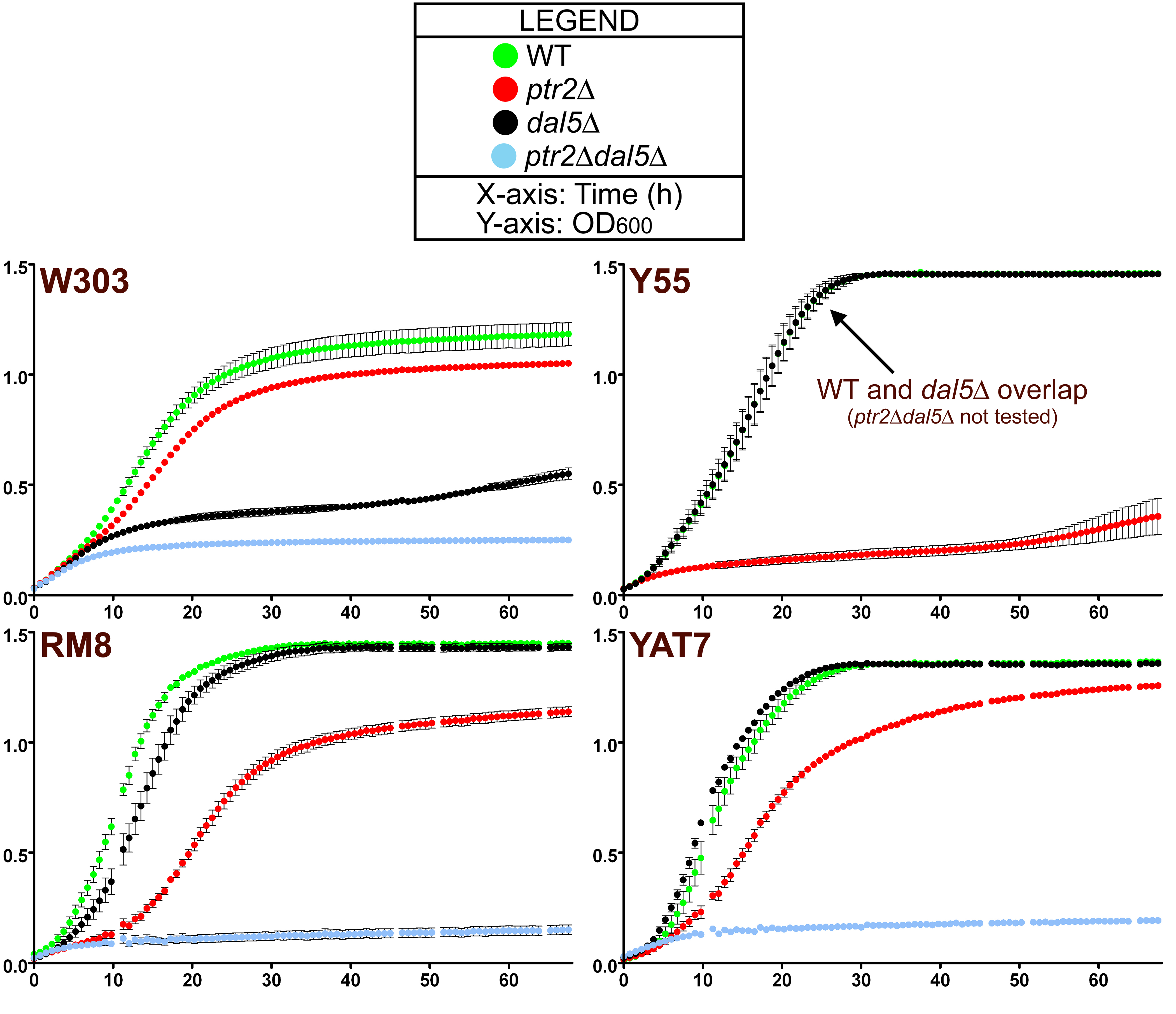

Supplement: Figure S1 — Wild-type, dal5Δ, ptr2Δ, and ptr2Δdal5Δ versions of the indicated strains were grown at 30 °C in liquid MM containing 2 mM Ala-Leu as the sole nitrogen source. A few time points were omitted because of technical difficulties with the Bioscreen C growth curve machine. (2.8 MB JPG) [file pgen.0010080.sg001.jpg]

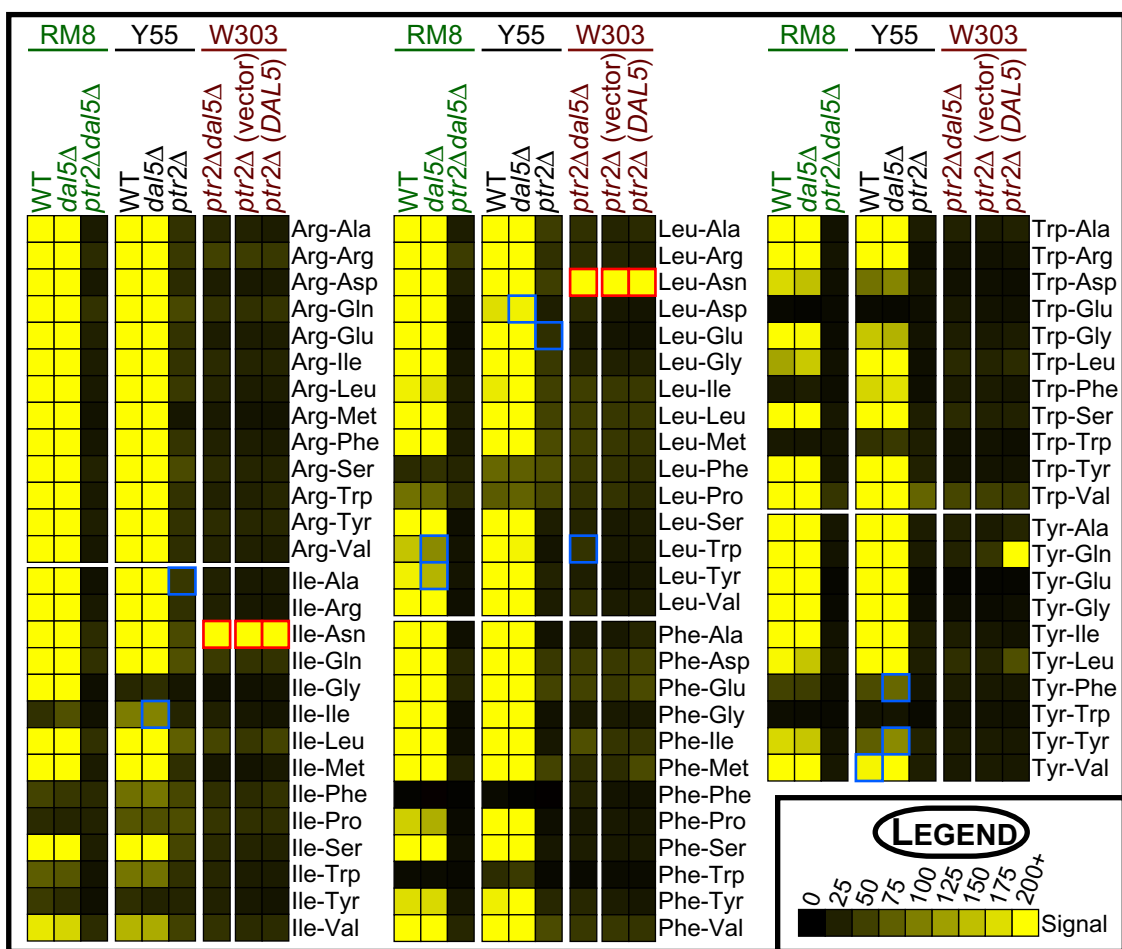

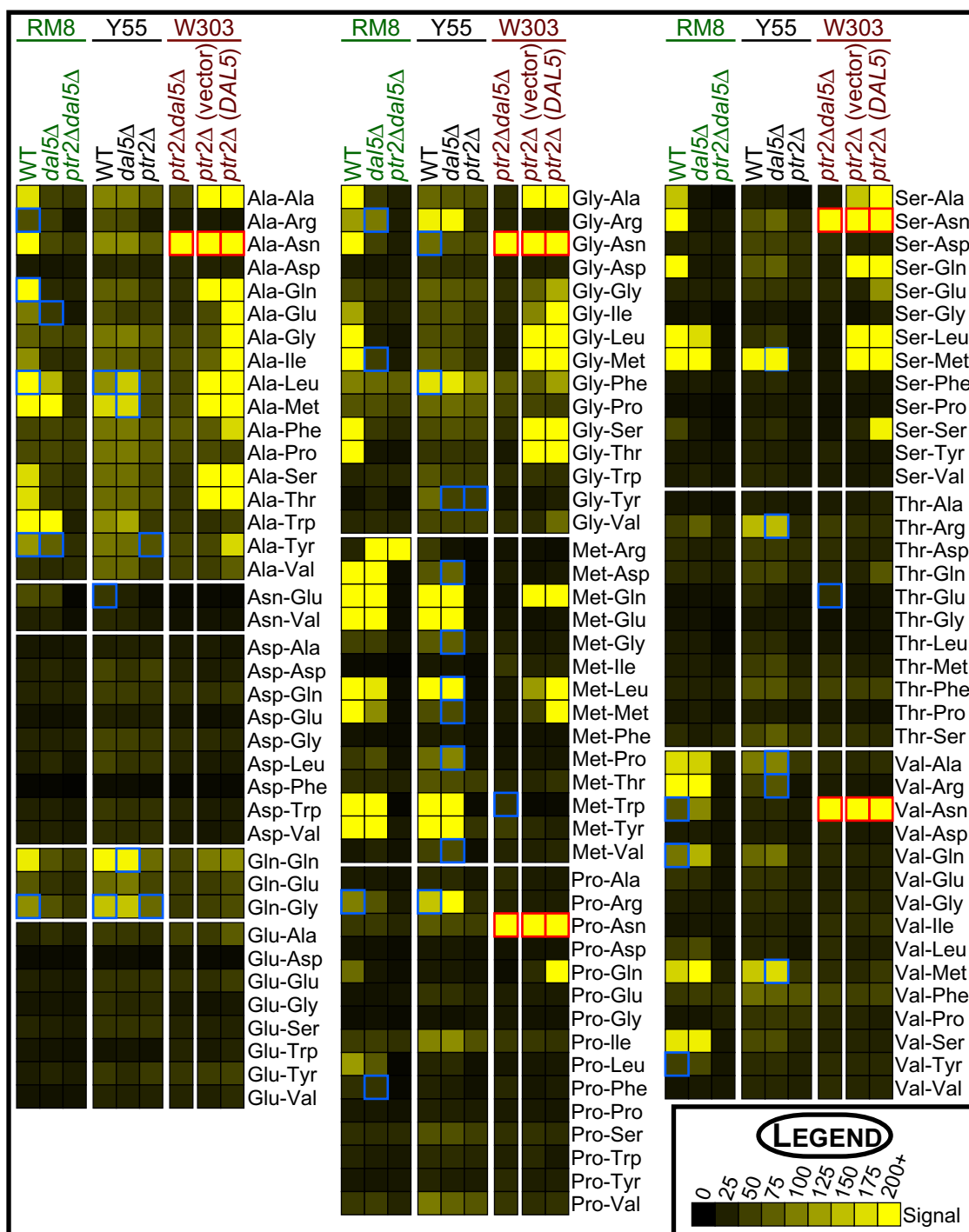

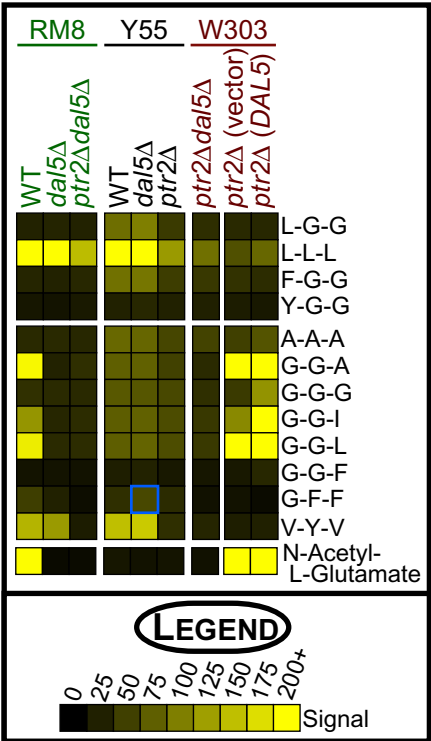

Supplement: Figure S2 — Refer to Figure 2 for details on data analysis and presentation. The column label ptr2Δ (vector) refers to strain W303-ptr2Δ transformed with the high-copy (2 μm) vector pRS426. The column label ptr2Δ (DAL5) refers to strain W303-ptr2Δ transformed with plasmid pRS426-DAL5. Note that the PM assays presented in this figure utilized a more recent manufacturing lot of the PM nitrogen plates than those presented in the other figures. Different lots can produce subtle differences in signal magnitude. The wild-type RM8 and Y55 PM data presented here were derived from the same lot to facilitate direct comparison. (631 KB PDF) [file pgen.0010080.sg002.pdf]
