# Supplementary material for: Harnessing Natural Diversity to Probe Metabolic Pathways
Source: PLoS Genet. 2005 Dec 30;1(6):e80. doi: 10.1371/journal.pgen.0010080 (PMC1342634; doi:10.1371/journal.pgen.0010080)
Supplement: Protocol S1 — The source and origins of the strains used in this study are indicated in Table 1. (39 KB DOC) [file pgen.0010080.sd005.doc]

**Protocol S1. Construction of the strains used in this study.** The source and origins of the strains used in this study are indicated in Table 1. To make all strains MATa *ura3*, some modifications were made, which are discussed below. Some of these strains were constructed at the same time as a panel of *hsc82*Δ mutants, and thus involve deletions and backcrosses that may seem unnecessary. All transformations were conducted using the standard lithium acetate technique [1].

Strains Y55 (also known as YAT3), YAT7, YAT17, and YAT21, were gifts of Dr. John McCusker. *URA3* was disrupted in these strains by short-flanking-homology (SFH) deletion [2,3] of the *URA3* region +46 to +759 using a small fragment of the plasmid pRS315 (ATCC#77144; [4]) amplified by the primers O-223 and O-224 (all primer sequences are provided in Table S1). Proper integration was verified by plating to 5-FOA and by colony PCR using primers O-138 and O-139.

YAT7 was further modified to eliminate an existing *kanMX3* disruption of the *HO* locus by making a diploid and then sporulating and screening for a MATa segregant sensitive to G418. The diploid was constructed using the *HO* expression plasmid YCp50-HO (a gift of Dr. Rochelle Esposito, University of Chicago), and the loss of the *kanMX3* cassette, presumably by looping out at the *MX* repeats, was verified using primers O-278 and O-279.

RM3 was modified by a series of steps, using the strains RM3-1a (MATa *leu2*Δ0 *ura3*Δ0 *ho*::*loxP-kanMX-loxP*) and RM3-1b ura3 (MATα *ura3*Δ0 *ho*::*lox*P*-kanMX-loxP*), which were obtained from Dr. Barbara Garvik and Dr. Robert Mortimer. First, *HSC82* was deleted from strain RM3-1a, utilizing a lox-flanked SFH construct amplified from plasmid pUG6-HygB by primers O-252 and O-253. The deletion was verified by colony PCR using primer pairs O-007+O-203 and O-008+O-204. The lox-flanked *kanMX* and *hphMX* cassettes were then excised using pSH47 [5] to express the Cre recombinase (making MATa *leu2*Δ0 *ura3*Δ0 *ho*::*loxP* *hsc82::loxP*). Excision of the *hphMX* cassette was confirmed using primers O-203 and O-204. This strain was then crossed to strain RM3-1b ura3 and sporulated to obtain a MATα *ura3*Δ0 *ho::loxP* spore, which was then crossed to a version of RM3-1a in which the lox-flanked *kanMX* cassette had been removed using pSH47. This diploid was then sporulated to obtain the final RM3 strain (MATa *ura3*Δ0 *ho*::*lox*P).

The original RM8 strain was obtained from B. Garvik/R. Mortimer (MATa *ura3*Δ0 *ho*::Kan-lox). The Kan cassette was excised as described for RM3.

The original strains obtained from B. Garvik for construction of the S288c strain were BY4712 (ATCC#200875; MATa *leu2*Δ0 *ho::loxP*), one of the designer deletion strains made from S288c, and 12447-16-2b (MATα *lys2*Δ0 *ura3*Δ0 *ho*::Kan-lox). *HSC82* was deleted from BY4712, as described for RM3, and the resulting strain was crossed to a variant of 12447-16-2b in which the Kan cassette had been excised by pSH47. From this cross, a spore (MATα *ura3*Δ0 *hsc82*::*loxP ho::loxP*) was isolated and backcrossed to BY4712. From this cross, a MATa *ura*3Δ0 *ho::loxP* spore was isolated, and designated S288c for use in this study.

The original W303 strain, designated 10556-23C (MATα *ura3-1* *can1-100* *ho*), was obtained from the laboratory of Dr. Gerald Fink. The mating type was switched and *HSC82* was deleted from this strain, as described for strain RM3. The strain was then backcrossed to 10556-23C and sporulated. A spore was chosen (MATa *ura3-1* *can1-100* *hsc82::loxP ho*) and again backcrossed to 10556-23C. The final strain (MATa *ura3-1* *can1-100* *ho*) was obtained from sporulation of this diploid.

*PTR2* was deleted with a SFH cassette amplified from plasmid pFA6a-GFP(S65T)-kanMX6 [6] (pAG32 [7] in the case of Y55) using primers O-286 and O-287 (the GFP portion of the plasmid was not amplified). Proper integration was verified using primer pairs O-007+O-288 and O-008+O-289. This deletion cassette replaced the *PTR2* region spanning +77 to +1781, relative to the start codon.

*CUP9* was deleted with a SFH cassette amplified from plasmid pFA6a-GFP(S65T)-kanMX6 using primers O-290 and O-291. Proper integration was verified using primer pairs O-007+O-292 and O-008+O-293. This deletion cassette replaced the *CUP9* region spanning +41 to +878, relative to the start codon.

*DAL5* was deleted with a *hphMX* marker from a SFH cassette amplified from pAG32 using primers O-327 and O-328. Proper integration was verified using primer pairs O-007+O-329 and O-008+O-330. This deletion cassette replaced the *DAL5* region spanning -70 to +1594, relative to the start codon.

Addition of a FLAG-2xGFP-6xHis tag to the C-terminus of endogenous *PTR2* was accomplished with a SFH cassette amplified from plasmid pPTR2-2xGFP-SFH-KanR using primers O-314 and O-315. Proper integration was ensured by selection on G418 media and verification by colony PCR using primer pair O-008+O-316. Final confirmation of proper integration was obtained by observation of membrane-localized GFP fluorescence in MMA+Leu media.

1. Ito H, Fukuda Y, Murata K, Kimura A (1983) Transformation of intact yeast cells treated with alkali cations. J Bacteriol 153: 163-168.

2. Baudin A, Ozier-Kalogeropoulos O, Denouel A, Lacroute F, Cullin C (1993) A simple and efficient method for direct gene deletion in Saccharomyces cerevisiae. Nucleic Acids Res 21: 3329-3330.

3. Wach A, Brachat A, Pohlmann R, Philippsen P (1994) New heterologous modules for classical or PCR-based gene disruptions in Saccharomyces cerevisiae. Yeast 10: 1793-1808.

4. Sikorski RS, Hieter P (1989) A system of shuttle vectors and yeast host strains designed for efficient manipulation of DNA in Saccharomyces cerevisiae. Genetics 122: 19-27.

5. Guldener U, Heck S, Fielder T, Beinhauer J, Hegemann JH (1996) A new efficient gene disruption cassette for repeated use in budding yeast. Nucleic Acids Res 24: 2519-2524.

6. Wach A, Brachat A, Alberti-Segui C, Rebischung C, Philippsen P (1997) Heterologous HIS3 marker and GFP reporter modules for PCR-targeting in Saccharomyces cerevisiae. Yeast 13: 1065-1075.

7. Goldstein AL, McCusker JH (1999) Three new dominant drug resistance cassettes for gene disruption in Saccharomyces cerevisiae. Yeast 15: 1541-1553.
