# Supplementary material for: Harnessing Natural Diversity to Probe Metabolic Pathways
Source: PLoS Genet. 2005 Dec 30;1(6):e80. doi: 10.1371/journal.pgen.0010080 (PMC1342634; doi:10.1371/journal.pgen.0010080)
Supplement: Protocol S2 — (25 KB DOC) [file pgen.0010080.sd006.doc]

**Protocol S2. Screen for Ptr2p-independent dipeptide utilization.** Screening was conducted using a pRS202-based genomic library (2µ;URA3) containing *Sau*3AI-digested genomic DNA with an average insert size of 6-8 kb (gift of Dr. Gerald Fink; originally constructed by Connelly and Hieter from a strain congenic with S288c [1]). Using standard lithium acetate transformation, ~1.3 µg of library DNA were transformed into ~1.2x10^8^ W303-*ptr2*Δ cells, which were then plated to 18 SD –ura plates and grown at 30 °C for ~40 h. Each plate contained a near-lawn (~30,000 colonies/plate), which was resuspended in sterile water and divided into six pools (*i.e.* three plates’ cells in each pool.) After washing twice with sterile water, ~1/10 of each pool was suspended in 50 ml of MM media lacking any nitrogen source and placed on a shaker for six hours. This step was included to reduce residual growth upon plating to the dipeptide media.

After growth in MM media for six hours, pools were split for use with screens on solid media (MM with 1 mM Ala-Leu as nitrogen source) and liquid media (MM with 0.75 mM Ala-Leu as nitrogen source). These Ala-Leu concentrations were chosen because they supported low colony size on solid media and no growth in liquid media. In the solid media screen, at least 2,000 cells were plated onto each of five plates for each pool, and the resulting ~60,000 colonies were screened for growth beyond the small colony size characteristic of W303-*ptr2*Δ containing a control vector. Of 11 colonies exhibiting enhanced growth, all were found to contain *PTR2* by diagnostic colony PCR using primers O-289 and O-314.

The liquid media screen was successful. After growth in the nitrogen-free MM media, cells from each pool were inoculated into MM media containing 0.75 mM Ala-Leu at OD_595_=0.015. From each pool, 300 µl aliquots were dispensed into 15 wells of a Bioscreen C plate, which was then incubated with heavy shaking at 30 °C for ~70 h. At this time, there were ~1-5 colonies growing at the bottom of each inoculated well (the shaking of the Bioscreen C is insufficient to keep late growth of individual cells in suspension.) From the wells of each pool, 16 colonies of varying size were each pipetted into 200 µl SD –Ura media in a 96-well plate. At this stage, although each of the 96 wells contained predominantly the cells from the pipetted colony, they likely also contained a small fraction of the originally inoculated, non-growing cells. To isolate colonies from an individual cell, 100x, 500x, and 1000x dilutions were made from the 96 wells and plated to SD –Ura plates using a bolt replicator. These plates were then grown for two days at 30 °C. One individual colony was isolated to represent each well.

These colonies were once more inoculated into MM media containing 0.7 mM Ala-Leu as nitrogen source and tested to verify the capacity for robust growth. Thirteen of the 96 isolates exhibited no, or very slow, growth. Seven of the isolates exhibited a capacity for growth that was not eliminated by loss of the plasmid by plating to 5-FOA media. Of the remaining 76 isolates, 73 contained *PTR2*, as verified by colony PCR. However, the three fastest-growing candidates conferred plasmid-dependent growth and did not appear to contain *PTR2*.

Plasmid rescue from the three non-*PTR2* candidates was conducted as follows. First, 2 ml of cells were grown overnight in SD –ura at 30 °C, washed, and resuspended in 200 µl of “breaking solution” (2% Triton X-100, 1% SDS, 100 mM NaCl, 1 mM EDTA, and 10 mM Tris(pH8.0).) Following addition of an equal volume of acid-washed glass beads, the mix was vortexed for 3 min at 4 °C. After two extractions with 25:24:1 PCI (phenol:chloroform:isoamyl alcohol), 0.5 µl of the remaining breaking solution was electroporated into 40 µl of 3x-diluted Invitrogen ELECTROMAX cells and resuspended in 2 ml LB media. After rotation at 37 °C for 1 hr, the electroporated cells were plated to LB+Amp and incubated at 37 °C overnight. Each electroporation yielded over 200 colonies, of which two were mini-prepped and retransformed into W303-*ptr2*Δ. Repetition of the growth assay verified that these plasmids all conferred robust growth when 0.7 mM Ala-Leu was supplied as sole nitrogen source.

The plasmids were then sequenced using primers O-206 and O-248, confirming that all three plasmids contained the same insert, spanning a region of Chromosome X from coordinates 716320 to 723802. This region contains the loci *YJR151W-A*, *DAL5*, and *PGU1*. Subcloning of the *DAL5* gene into pRS426 (making pRS426-*DAL5*) confirmed that the *DAL5* gene confers the phenotype.

1. Connelly C, Hieter P (1996) Budding yeast SKP1 encodes an evolutionarily conserved kinetochore protein required for cell cycle progression. Cell 86: 275-285.
