# Supplementary material for: Harnessing Natural Diversity to Probe Metabolic Pathways
Source: PLoS Genet. 2005 Dec 30;1(6):e80. doi: 10.1371/journal.pgen.0010080 (PMC1342634; doi:10.1371/journal.pgen.0010080)
Supplement: Table S1 — (28 KB DOC) [file pgen.0010080.st001.doc]

**Table S1. Polymorphisms in the region upstream of *PTR2*, relative to the standard**

S288c sequence

| Strain | C-799T | C-582T | A-555G | A-496G | T-458G | G-385C | T-297C | T-152C | ins-46T | del-32CTT |
| --- | --- | --- | --- | --- | --- | --- | --- | --- | --- | --- |
| S288c | -- | -- | -- | -- | -- | -- | -- | -- | -- | -- |
| W303 | + | -- | -- | -- | -- | -- | -- | -- | -- | -- |
| RM8 | + | + | + | + | -- | + | + | + | -- | -- |
| YAT7 | -- | -- | -- | + | + | -- | -- | -- | +*^a^* | +*^b^* |

A region encompassing the bases spanning from positions -899 to +3 relative to the *PTR2* stop codon was sequenced in the indicated strains.

*^a^* “ins-46T” refers to the expansion of a string of 11 “T”’s spanning from -46 to -36 by an additional “T”.

*^b^* “del-32CTT” refers to a deletion of the bases “CTT” spanning from -32 to -30 of the published S288c sequence.
