# Supplementary material for: Harnessing Natural Diversity to Probe Metabolic Pathways
Source: PLoS Genet. 2005 Dec 30;1(6):e80. doi: 10.1371/journal.pgen.0010080 (PMC1342634; doi:10.1371/journal.pgen.0010080)
Supplement: Table S2 — (52 KB DOC) [file pgen.0010080.st002.doc]

**Table S2**. Oligos used in this study

| Oligo | Sequence | Use |
| --- | --- | --- |
| O-007 | CAATTCAACGCGTCTGTGAGG | Verify SFH deletions |
| O-008 | GATTCTTGTTTTCAAGAACTTG | Verify SFH deletions |
| O-138 | TATACGCATATGTGGTGTTG | Verify *URA3* deletion |
| O-139 | CGACCGAGATTCCCGGGTAA | Verify *URA3* deletion |
| O-203 | ATGGTTTGTGAACAGATTGA | Verify *HSC82* deletion |
| O-204 | TTGAATGGACGCCGAATTGC | Verify *HSC82* deletion |
| O-206 | GTATCGATAAGCTTGATATC | Sequencing genomic library plasmid insert |
| O-223 | ATGTCGAAAGCTACATATAAGGAACGTGCTGCTACTCATCCTAGT  TTACCAATGCTTAATCAGTGAG | Amplify *URA3* SFH deletion cassette |
| O-224 | TTAGTTTTGCTGGCCGCATCTTCTCAAATATGCTTCCCAGCCTGC  GCAACAACGTTGCGCAAACTAT | Amplify *URA3* SFH deletion cassette |
| O-248 | TAATACGACTCACTATAGGG | Sequencing genomic library plasmid insert |
| O-252 | GTGGCATGTGAACTGCCTACCGTAAGTGACATGAACACATGCATT  CAGCTGAAGCTTCGTACGCTGC | Amplify lox-flanked *HSC82* SFH deletion cassette |
| O-253 | GGCGAAAAAAGAGATTAATCAACTTCTTCCATCTCGGTGTCAGCT  GCATAGGCCACTAGTGGATCTG | Amplify lox-flanked *HSC82* SFH deletion cassette |
| O-278 | CGCAAGTCCTGTTTCTATGC | Verify KanMX excision from *HO* |
| O-279 | CTACGTTGCCTCCATCG | Verify KanMX excision from *HO* |
| O-286 | CAGGACGAAAAGCAAGGCGACTTCCCGGTCATCGAAGAGGGCCTC  GTCCCCGCCGGGTCA | Amplify *PTR2* SFH deletion cassette |
| O-287 | ACTTTCCATTGGTTCTAATATTTCAATATCGTTAGCTTTACACTG  GATGGCGGCGTTAGT | Amplify *PTR2* SFH deletion cassette |
| O-288 | GGGCTAACGGCGTCCGCAGA | Verify *PTR2* deletion |
| O-289 | AAGAGAAAGTGTGGTCACAC | Verify *PTR2* deletion |
| O-290 | ATGAATTATAACTGCGAAATACAAAACAGGAACAGTAAGAGCCTC  GTCCCCGCCGGGTCA | Amplify *CUP9* SFH deletion cassette |
| O-291 | ATTCATATCAGGGTTGGATAGCTTTTTCAATTCTTCCAGCCACTG  GATGGCGGCGTTAGT | Amplify *CUP9* SFH deletion cassette |
| O-292 | GCGTGCTTCCTCACACTGGC | Verify *CUP9* deletion |
| O-293 | GAAAGAGAAGATGATAACTA | Verify *CUP9* deletion |
| O-314 | CGAATTTGATCTCAATCCAATTTCCGCACC | SFH integration of FLAG-2xGFP-6xHIS/kanMX4 at C-terminus of *PTR2* |
| O-315 | AATATGGACCTCTCTTCACAAATTGTTCTATAATACAATATATAT  TCTGGGCAGATGATGTCGAG | SFH integration of FLAG-2xGFP-6xHIS/kanMX4 at C-terminus of *PTR2* |
| O-316 | ATTGATCTTTGCTGGTAAGC | Verify FLAG-2xGFP-6xHIS/KanR integration |
| O-321 | ATCTGAGCCTTGGCTGGGAT | *PTR2* promoter sequencing (+30 to +11) |
| O-322 | CCGTCCTTTTCACTTCACGT | *PTR2* promoter sequencing (-466 to -447) |
| O-323 | CCGTACAGATAAGAAACCCA | *PTR2* promoter sequencing (-368 to -387) |
| O-324 | GATAAGCTTGGGGTAACGCA | *PTR2* promoter sequencing (-897 to -878) |
| PTR2-FLAG-GFP-F | GATCCACCACCAAATATGACTACAAGGACGACGATGACAAGCTTG  ATATCAACAAGACAGGG | Amplify GFP for insertion into pMS2 |
| PTR2-FLAG-GFP-R | GATGATGATGACTGCTGCCGCTGCCGCGCGGCACACCGGT  CCTCGAGCCGCTTTTCTTGTAC | Amplify GFP for insertion into pMS2 |
